# Full in-frame exon 3 skipping of *BRCA2* confers high risk of breast and/or ovarian cancer

**DOI:** 10.18632/oncotarget.24671

**Published:** 2018-04-03

**Authors:** Sandrine M. Caputo, Mélanie Léone, Francesca Damiola, Asa Ehlen, Aura Carreira, Pascaline Gaidrat, Alexandra Martins, Rita D. Brandão, Ana Peixoto, Ana Vega, Claude Houdayer, Capucine Delnatte, Myriam Bronner, Danièle Muller, Laurent Castera, Marine Guillaud-Bataille, Inge Søkilde, Nancy Uhrhammer, Sophie Demontety, Hélène Tubeuf, Gaïa Castelain, French COVAR group collaborators, Uffe Birk Jensen, Ambre Petitalot, Sophie Krieger, Cédrick Lefol, Virginie Moncoutier, Nadia Boutry-Kryza, Henriette Roed Nielsen, Olga Sinilnikova, Dominique Stoppa-Lyonnet, Amanda B. Spurdle, Manuel R. Teixeira, Florence Coulet, Mads Thomassen, Etienne Rouleau

**Affiliations:** ^1^ Institut Curie, Service de Génétique, Paris, France; ^2^ Unité Mixte de Génétique Constitutionnelle des Cancers Fréquents, Hospices Civils de Lyon/Centre Léon Bérard, Lyon, France; ^3^ Centre Léon Bérard, Lyon, France; ^4^ Institut Curie, PSL Research University, CNRS UMR3348, Orsay, France; ^5^ Université Paris Sud, Université Paris-Saclay, CNRS UMR3348, Orsay, France; ^6^ Inserm-U1245, UNIROUEN, Normandie University, Normandy Centre for Genomic and Personalized Medicine, Rouen, France; ^7^ Maastricht University, Maastricht, Netherlands; ^8^ Department of Genetics, Portuguese Oncology Institute, Porto, Portugal; ^9^ Fundación Pública Galega de Medicina Xenómica-SERGAS, Grupo de Medicina Xenómica-USC, CIBERER, IDIS, Santiago de Compostela, Spain; ^10^ Université Paris Descartes, Paris, France; ^11^ Service de Génétique médicale, CHU Nantes, Nantes, France; ^12^ Service de Génétique, CHU Nancy Brabois, Nancy, France; ^13^ Laboratoire d’Oncogénétique, Centre Paul Strauss, Strasbourg, France; ^14^ Laboratoire de biologie et de génétique du cancer, CLCC François Baclesse, INSERM 1079 Centre Normand de Génomique et de Médecine Personnalisée, Caen, France; ^15^ Institut Gustave Roussy, Villejuif, France; ^16^ Section of Molecular Diagnostics, Department of Clinical Biochemistry, Aalborg University Hospital, Aalborg, Denmark; ^17^ Laboratoire de Biologie Médicale, CLCC Jean Perrin, Clermont-Ferrand, France; ^18^ Department of Clinical Genetics, Aarhus University Hospital, Aarhus, Denmark; ^19^ Department of Clinical Genetics, Vejle Hospital, Odense, Denmark; ^20^ Genetics and Comp utational Biology Division, QIMR Berghofer Medical Research Institute, Herston, Brisbane, Australia; ^21^ Institute of Biomedical Sciences, University of Porto, Porto, Portugal; ^22^ Laboratoire d’Oncogénétique et d’Angiogénétique Moléculaire, Groupe Hospitalier Pitié-Salpêtrière, Paris, France; ^23^ Department of Clinical Genetics, Odense University Hospital, Odense, Denmark; ^24^ Interactive Biosoftware, Rouen, France

**Keywords:** BRCA2 exon3, PALB2, variants, RNA splicing defects, splice donor site

## Abstract

Germline pathogenic variants in the *BRCA2* gene are associated with a cumulative high risk of breast/ovarian cancer. Several *BRCA2* variants result in complete loss of the exon-3 at the transcript level. The pathogenicity of these variants and the functional impact of loss of exon 3 have yet to be established.

As a collaboration of the COVAR clinical trial group (France), and the ENIGMA consortium for investigating breast cancer gene variants, this study evaluated 8 *BRCA2* variants resulting in complete deletion of exon 3. Clinical information for 39 families was gathered from Portugal, France, Denmark and Sweden. Multifactorial likelihood analyses were conducted using information from 293 patients, for 7 out of the 8 variants (including 6 intronic). For all variants combined the likelihood ratio in favor of causality was 4.39*10^25^. These results provide convincing evidence for the pathogenicity of all examined variants that lead to a total exon 3 skipping, and suggest that other variants that result in complete loss of exon 3 at the molecular level could be associated with a high risk of cancer comparable to that associated with classical pathogenic variants in *BRCA1* or *BRCA2* gene. In addition, our functional study shows, for the first time, that deletion of exon 3 impairs the ability of cells to survive upon Mitomycin-C treatment, supporting lack of function for the altered BRCA2 protein in these cells.

Finally, this study demonstrates that any variant leading to expression of only *BRCA2* delta-exon 3 will be associated with an increased risk of breast and ovarian cancer.

## INTRODUCTION

The *BRCA2* gene (MIM#600185) is a tumor suppressor gene that codes for a 3,418 amino-acid protein discovered in 1995 [1]. BRCA2 is involved in the maintenance of genome integrity by means of two main functions: DNA repair by homologous recombination and stabilization of replication forks under replication stress [2–7]. Pathogenic germline *BRCA2* variants predispose to high risk of breast and ovarian cancer and are associated with the Hereditary Breast and Ovarian Cancer syndrome (HBOC) [8, 9].

The cancer risk for *BRCA2* pathogenic variant carriers is 55% for breast cancer, 16.5% for ovarian cancer, and 62% for contralateral breast cancer [10]. The variants identified in women are mostly classified as pathogenic when they lead to a premature translation termination (premature stop codon). However, variant classification is complicated toward the related risk of cancer when the molecular or functional effect of a variant is unclear [11]. A recent study showed that the cancer risk of pathogenic variant carriers in the different regions of *BRCA2* is not similar. *BRCA2* pathogenic variants localized in 5’ (5’ to c.2830) and 3’ (3’ to c.6402) regions were associated with a significant higher breast cancer risk compared with pathogenic variants within the central region [12].

To date, several functional domains have been described in BRCA2 including the C-terminal DNA binding domain [13]; the BRC repeats in the central region of the protein have a well-established function in the interaction with RAD51 [14–16]. The N-terminal region has been less extensively explored, but it has recently been shown to contain a second DNA binding domain [17]. The N-terminal region of BRCA2 also comprises exon 3, amino acids 23 to 105. According to the literature, exon 3 is found to be bipartite with a primary activating region (PAR: aa 23-60) and an auxiliary activating region (AAR: aa 60-105). The AAR region has little homology with c-Jun [18] and would be responsible for a kinase activity different from that of c-Jun or independently of the JNK signaling pathway [19, 20]. Milner et al. have shown that these residues bind specifically as a kinase. In addition, the team of Lin et al. tested the possible phosphorylation of BRCA2 by PLK1 in this region [21]. The primary activating region (PAR) has an activation capacity and is responsible for protein-protein interaction. These residues are involved in an interaction with EMSY, but with no obvious function [22]. EMSY has endogenous transcriptional repressor activity, and participates in DNA damage foci formation. In 2002, Preobrazhenska et al. showed that BRCA2 (exon 3) is also a Smad-interacting protein which synergizes with Smad3 in activation of gene expression [23].

Most interestingly, the PAR domain also interacts with the PALB2 protein (Partner and localizer of BRCA2) [24]. PALB2 is involved in DNA repair by homologous recombination and forms a complex with BRCA1 and BRCA2 [25].

In the literature, several variants within exon 3 have been described with partial splicing effect (c.68-7T>A, c.68-7_8delinsAA, c.68-7delT) or total splicing effect (c.316+4del, c.156_157insAlu, for example) and considered as neutral (c.68-7T>A, c.125A>G) or causal (c.156_157insAlu) [26, 27]. Furthermore, although point mutations and large rearrangements in the *BRCA2* gene giving rise to exon 3 skipping at the mRNA level might be associated to the abnormal phenotype in breast/ovarian cancer families, the skipping of exon 3 does not alter the reading frame, and the RAD51 binding sites, nuclear localization signals in the 3’ region of BRCA2 or other functional domains of the protein still remain intact. Therefore, the heterogeneity of splicing effects with the expression of low quantity of full transcript has shed the doubt on the pathogenicity. Several variants of *BRCA2* are known to result in total loss of exon 3 at the transcript level. For example, the variant with the Alu insertion in the middle of exon 3 (c.156_157insAlu) have been reported in families of Portuguese ancestry and could be due to the existence of co-founding variants related to this insertion, as all families have the same ancestry [27–29]. The c.68-?_316+?del and c.316+4del (previously reported as c.316+3delA) variants with loss of exon 3 tend to increase breast cancer risk in the affected families [26]. For those variants in these studies, the number of families and variants were not sufficient for detailed cancer risk assessment, using co-segregation and other clinical information.

Moreover, the functional role of the affected delta-3 BRCA2 protein domain has not been established and the existence of a stable delta-3 BRCA2 protein, although theoretically possible, has not been proven. Several variants lead to a complete *in frame* deletion of exon 3 has been identified in different countries. A study of the impact of variants leading to transcripts that encode full delta-3 BRCA2 was conducted in France within the framework of the COsegregation of VARiants in the *BRCA1/2* Genes (COVAR) clinical trial [30]. In parallel, the ENIGMA (Evidence-Based Network for the Interpretation of Germline Mutant Alleles) international consortium (including members of COVAR), which is an initiative to evaluate risk and determine the clinical significance of unclassified *BRCA1*, *BRCA2* and other breast cancer susceptibility genes variants, has conducted a collaborative study of known or potentially spliceogenic *BRCA2* variants [31]. To increase the statistical power and reduce the risk of population bias we have included families from both initiatives in the present analysis.

This study addressed several questions related to eight *BRCA2* variants reported to cause complete loss of exon 3 at the transcriptional level that are expected to lead to a delta-3 protein. First, we selected and confirmed the variants exclusively leading to full skipping of this exon to avoid any biais related to partial expression. Second, we performed functional analysis to determine the impact of a delta-exon-3 BRCA2 protein on BRCA2 function. Finally, we estimated for each variant the causality and probability of being deleterious using multifactorial likelihood analysis including, in addition to segregation data, other clinical estimates of variant pathogenicity.

## RESULTS

### Selection of variants to address the pathogenic nature of complete loss of *BRCA2* exon 3

In this study, we selected a total of 8 *BRCA2* variants including 6 genetic changes for which there was evidence from patient RNA data that they were associated with total in-frame exon 3 skipping (Supplementary Table 1A). In addition, we included 2 other variants identified in our cohort located at the invariant positions of the 5’ splice site of *BRCA2* exon 3 (IVS3+1/+2), expected to cause the same effect.

### Confirmation of full exon skipping induced by variants mapping at the 5’ splice site of *BRCA2* intron 3

**Figure 1:**
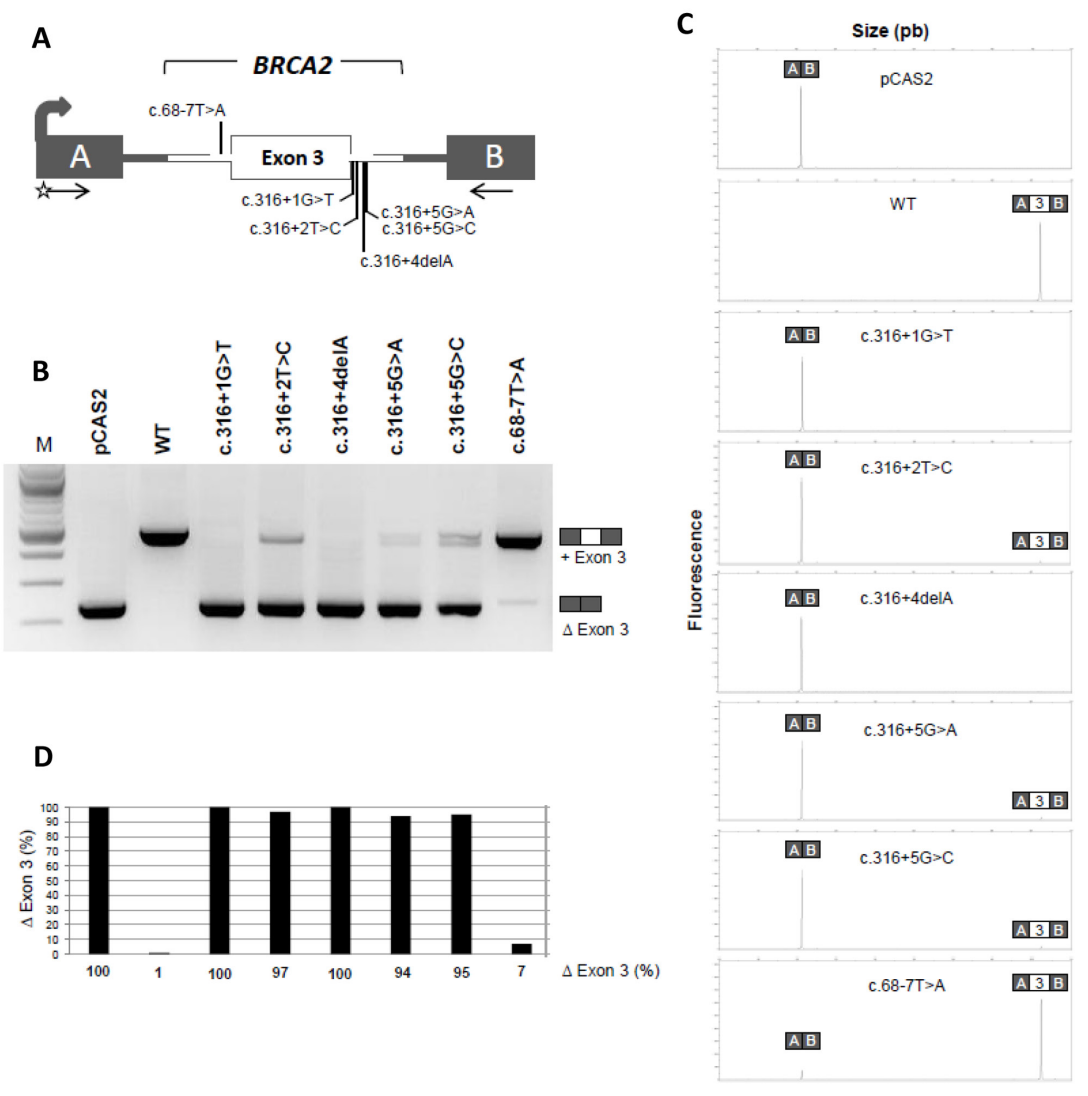
A minigene splicing assay confirms that variants located at the 5’ splice site of *BRCA2* exon 3 induce drastic exon skipping **(A)** Structure of the pCAS2-BRCA2-exon 3 minigene used in the minigene splicing assay. The grey arrow indicates the CMV promoter, boxes represent exons, lines in between indicate introns, and arrows under the exons represent primers used in RT-PCR reactions. The positions of the variants analyzed in the minigene assay are also indicated. The minigenes were generated by inserting a genomic fragment containing *BRCA2* exon 3 and part of flanking intronic sequences into the intron of pCAS2 (either by using the proband’s gDNA as template or by introducing the variants into the minigenes by site-directed mutagenesis). WT and mutant constructs, as indicated, were then transfected into HeLa cells and the minigene transcripts were analyzed by RT-PCR, as described in Materials and Methods. **(B)** Analysis of the splicing pattern of the pCAS2-BRCA2-exon 3 minigenes carrying the variants of interest. *BRCA2* c.68-7T>A was used as control. The image shows the results of a representative experiment, in which the RT-PCR products were separated on a 2.5% agarose gel stained with ethidium bromide and visualized by exposure to ultraviolet light. M, size marker (100 bp DNA ladder, New England Biolabs). The identities of the two major RT-PCR products, with or without exon 3, are indicated on the right. **(C)** Representative results from fluorescent RT-PCR reactions (equivalent to those shown in B) performed by using a fluorescent forward primer and then separated under denaturing conditions by capillary electrophoresis on an automated sequencer. The identities of the RT-PCR products are shown above the peaks. **(D)** Level of exon 3 skipping observed in the minigene assay as determined by fluorescent RT-PCR (variants displayed in the same order as in B). Results are shown as the average of three independent experiments and are expressed as percentage of exon skipping (exon skipping product x 100/total transcripts).

To evaluate the impact on splicing of the 5 variants located at the 5’ splice site of *BRCA2* exon 3, we performed a cell-based minigene assay. As shown in Figure 1, the pCAS2-BRCA2 exon 3 wild-type minigene led to the major production of transcripts containing exon 3 (99% inclusion), whereas the minigene carrying the *BRCA2* c.68-7T>A variant, used here as a control, induced weak exon skipping (7%), as previously shown in patient RNA [26]. In contrast, *BRCA2* c.316+1G>T and c.316+4del were responsible of total exon 3 skipping (100%), as quantified by fluorescent RT-PCR. The 3 other intronic variants tested, *BRCA2* c.316+2T>C, c.316+5G>A and c.316+5G>C, induced major, quasi-complete, exon 3 skipping (respectively, 97%, 94% and 95%). These results are in agreement with patient RNA data (Supplementary Table 1A) and/or *in silico* predictions (Supplementary Table 1B).

### Impact in the function of BRCA2

**Figure 2:**
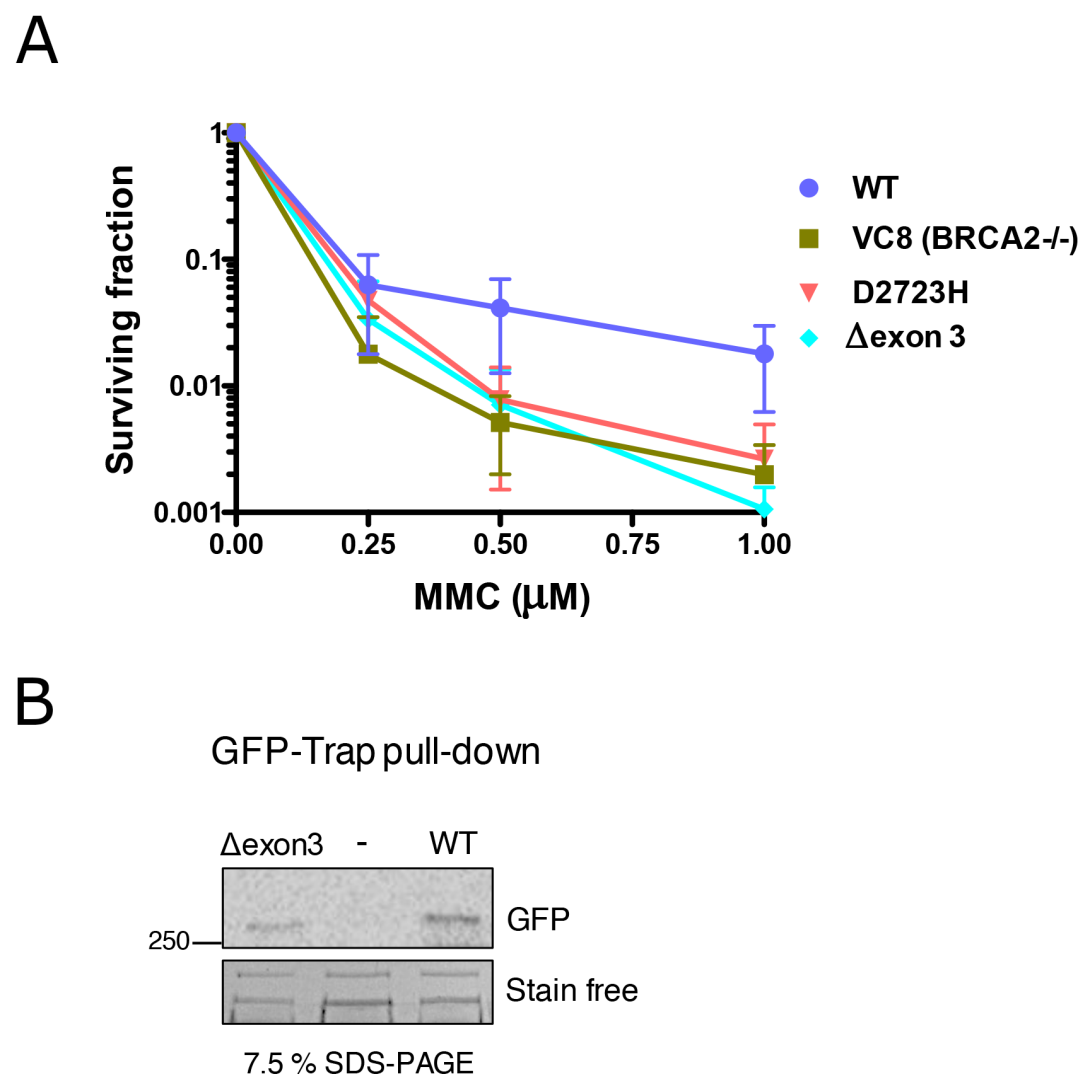
Hypersensitivity of cells exposed to Mitomycin C (MMC) expressing BRCA2 cDNA carrying Δexon 3 **(A)** Clonogenic survival assay of VC8 cells (Brca2 -/-) expressing human BRCA2 wild-type (WT), D2723H (c.8167G>C, exon 18) missense mutation or Δexon 3 in response to the indicated concentrations of MMC. Error bars, S.D. (n=3). **(B)** Western blot showing GFP-BRCA2 protein immunoprecipitated from the cell population used for seeding for the clonogenic survival assay (A). StainFree imaging of the gel before transfer was used as a loading control (only a cropped image of the gel is shown).

To investigate the functional impact of the complete deletion of exon 3, we generated a cDNA expressing construct carrying this deletion. As expected, we found that GFP-MBP-BRCA2 WT fully complemented *brca2*-deficient hamster cells (VC8) [32]. In contrast, the complete deletion of exon 3 of BRCA2 rendered cells hypersensitive to Mitomycin C (MMC) treatment to the same level as the known pathogenic *BRCA2* variant D2723H (c.8167G>C, p.Asp2723His, exon 18) or *Brca2*-deficient cells (Figure 2A). The deletion of exon 3 did not affect the translation and stability of the protein, as it was readily detected by Western Blot (Figure 2B). These results strongly suggest that the region encoded by exon 3 is necessary to restore cell viability following MMC-induced DNA damage.

### Causality score from multifactorial likelihood analysis, including segregation data

Multifactorial likelihood analyses were conducted using information from 293 patients (194 confirmed as carriers of the variant under study) in 26 families. Variants were categorized based on the final posterior probability, according to the classification system for sequence variants proposed by the 2008 IARC working group on unclassified genetic variants [33].

**Figure 3:**
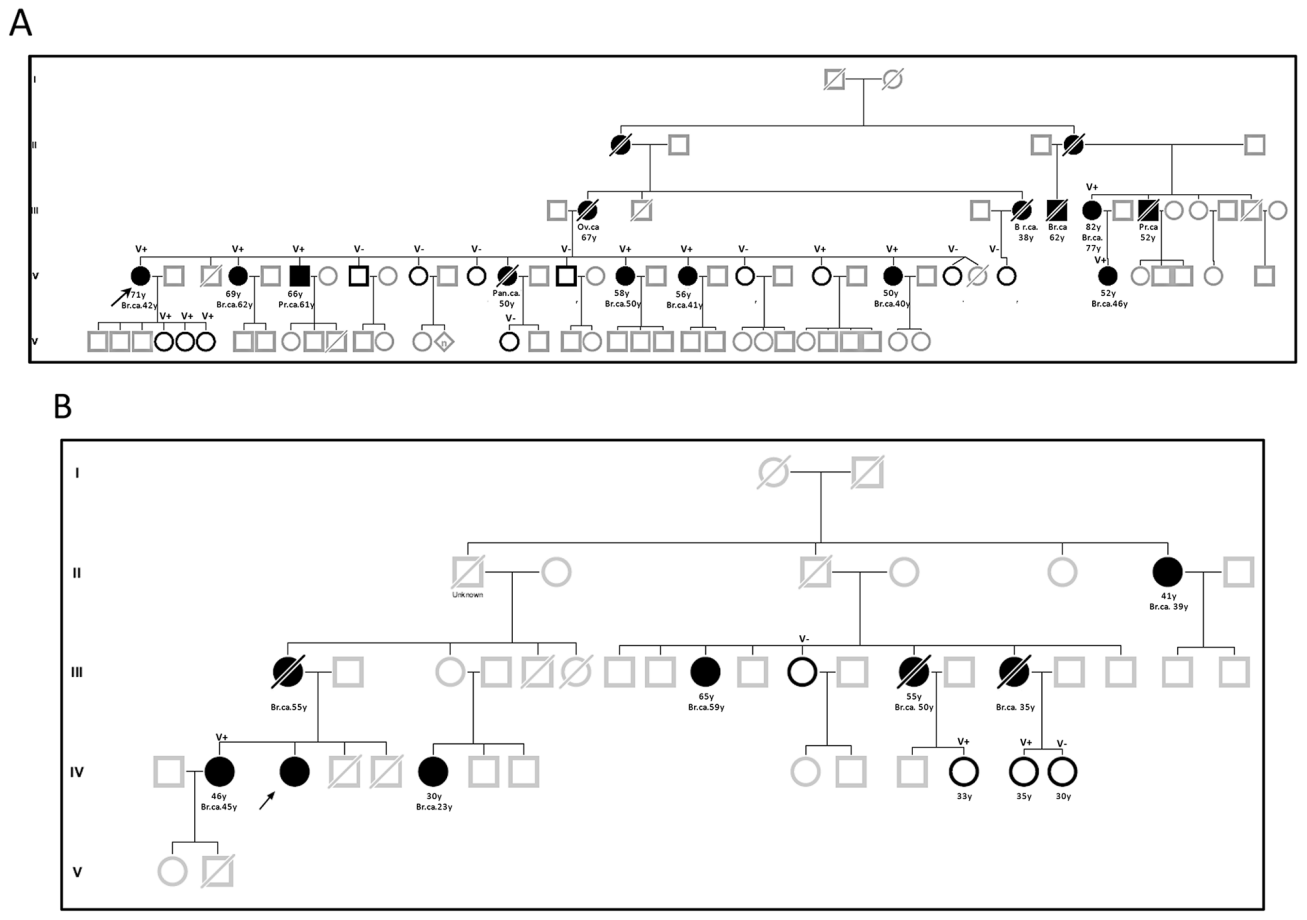
Pedigrees of families with *BRCA2* c. 316+5G>C **(A)** and c.156_157insAlu **(B)** variants. Circles indicate females and squares indicate males, in bold relatives with mutated genetic status known but not affected. Slashes indicate death. The proband is indicated by an arrow. Current ages or age at death and age at cancer diagnosis are listed below each individual, together with genetic status when known.

The co-segregation analysis for variants *BRCA2* c.316+5G>C and c.156_157insAlu were the most informative, with 10 and 13 informative families, respectively, and with a substantial segregation likelihood score of 14544.25 and 6.4124x10^12^ respectively (each with a highly informative family, Figure 3). *BRCA2* c.68-?_316+?del also presented a strong segregation likelihood score (1393.30) from only one family with 15 variant carriers among 19 individuals tested. LR from segregation data alone was in favor of pathogenicity for all other variants with data: c.277_317-726delinsCCAT (19.35), c.316+5G>A (3.69) and c.316+4del (86.77).

Breast tumor pathology data were available for some variant carriers (Supplementary Table 2). The tumor was estrogen receptor-positive and progesterone receptor-negative. For c.316+5G>A, the proband presented breast cancer diagnosed at the age of 30 years that was estrogen receptor-positive. For c.316+5G>C, we present pathology data for 8 patients: age of onset of breast cancer was between 25 and 55 years, with minimum grade 2 and positive estrogen receptor status in almost all cases (6/7). For c.156_157insAlu, pathology data were available for 25 patients: ages of onset of breast cancer were between 28 and 66 years and estrogen receptor status was positive for almost all tumors. LR based on pathology information was higher than 1 for all variants for which relevant information was available.

Pathogenicity was assessed for each variant individually, and also for all 7 variants for which information was available, as they were considered to have resulted from the same RNA and protein event. As shown in Table 1, when considering variants individually, three variants had posterior probabilities >0.99 that categorized them as pathogenic (class 5) providing convincing evidence that these variants are associated with a cancer risk equivalent to the average (mostly truncating) pathogenic variants in *BRCA2*. For all variants combined, the likelihood ratio in favour of causality was 4.39*1025, and the posterior probability of pathogenicity was 1.00.

**Table 1:** Classification based on multifactorial likelihood analysis for *BRCA2* variants leading to exon 3 deletion at the mRNA level

HGVS DNA nomenclature c.	RNA impact	Number of families	Number of families for cosegregation analysis	Number of relatives for cosegregation analysis (carriers)	LR cosegregation	LR Pathology	Combined LR causality	Prior probability [67]	Posterior probability	Class
**c.68_316del**	full skipping	1	1	19 (15)	1393,30	-	1393,30	0,5	0,999282796	Class 5
**c.156_157insAlu**	full skipping	20	18	179 (114)	6,4124E+12	5,23	3,35E+13	0,5	1	Class 5
**c.277_317-726delinsCCAT**	full skipping	1	1	5 (5)	19,35	1,06	20,51	0,5	0,953504137	Class 4
**c.316+1G>T**	full skipping	1	1	7 (4)	3,43	-	3,43	0,5	0,774439482	Class 3
**c.316+2T>C**	full skipping	2	0	0	-	-	-	-	-	-
**c.316+4del**	full skipping	2	1	15 (10)	86,77	-	86,77	0,5	0,988606469	Class 4
**c.316+5G>A**	full skipping	2	1	6 (5)	3,69	1,15	4,24	0,5	0,809128615	Class 3
**c.316+5G>C**	full skipping	12	10	62 (41)	14544,25	2,94	36332,99	0,5	0,999976616	Class 5
**Score total for all variants**	full skipping	41	33	293 (194)			4,39E+25	0,5	1	Class 5

## DISCUSSION

Clinical classification of sequence variants is often limited by the small number of families or the lack of clinical information. This study shows the potential of combined analysis of several variants causing the same functional effect to increase the power of multifactorial likelihood analysis, in particular co-segregation data. In this study, we combined all known variants resulting in complete loss of exon 3, including 5 intronic changes directly affecting the splice donor site as confirmed by splicing assay. This hypothesis was first established with the analysis of the large rearrangement of exon 3 [26]; however, the classification of complete deletions of exon 3 in *BRCA2* in the clinical setting has been controversial [26, 28, 29, 34–38]. In this study, we definitively prove that a full total deletion of exon 3 of BRCA2 is pathogenic.

Indeed, in this study, we validate the full in-frame exon 3 skipping for six *BRCA2* variants with the minigene approach avoiding any ambiguity on a partial expression of a full transcript. This approach was required to gather several variants with a putative similar impact on splicing and to distinguish those variants to those on the intron 2 (c.68-7A>T).

Then this study presents results from multifactorial likelihood analysis of six out of eight *BRCA2* variants proven to lead to a full in-frame exon 3 skipping at the transcriptional level, including co-segregation analysis of multiple families (c.68-?_316+?del, c.156_157insAlu, c.316+1G>T, c.316+4del, c.316+5G>A, c.316+5G>C). There was sufficient evidence to classify individually three variants as pathogenic (Class 5). Notably, the likelihood ratio of causality from segregation data alone was 1393:1 from a single family carrying c.68-?_316+?del, 14544:1 for 12 families carrying c.316+5G>C, and 6.4x10^12^ for 13 families carrying c.156_157insAlu. Another variant (c.316+4del) had sufficient information available to place it as likely pathogenic (Class 4). All evidence from segregation and pathology data for individual variants, irrespective of their individual classification, was in favor of pathogenicity. The likelihood ratio in favor of causality was 4.39*10^25^, and the posterior probability of pathogenicity was 1.00 for all variants combined. These results provide convincing evidence for the pathogenicity of all seven variants that lead to complete deletion of exon 3, and suggest that other variants that result in complete loss of exon 3 at the molecular level will be associated with a high risk of cancer comparable to other classical pathogenic variants in *BRCA2* (largely truncating), including c.316+2G>T. The pathology of consecutive breast cancers related to those variants were estrogen receptor-positive as typically described for *BRCA2* breast tumors (Supplementary Table 2; 29/35 ER-positive, 32/35 PR-positive, 4/18 HER2-positive, 10/25 grade 2) [39]. The variant c.277_317-726delinsCCAT from Nordling et al. presented one family with pathology data for one carrier with invasive cancer of the left breast and multiple axillary and supraclavicular node metastases at the age of 41 years [32]. These findings are of direct relevance for counseling and management of individuals found to carry these variants.

Importantly, our clinical analysis is fully supported by functional assays demonstrating that ectopically expressed BRCA2 transcripts lacking exon 3 confer hypersensitivity to DNA damage (MMC treatment). Our functional study shows, for the first time, that deletion of exon 3 impairs the ability of cells to survive MMC treatment, strongly suggesting that BRCA2 is not functional in these cells. These findings correlate with an increased cancer risk as calculated in our multifactorial likelihood analysis.

**Figure 4:**
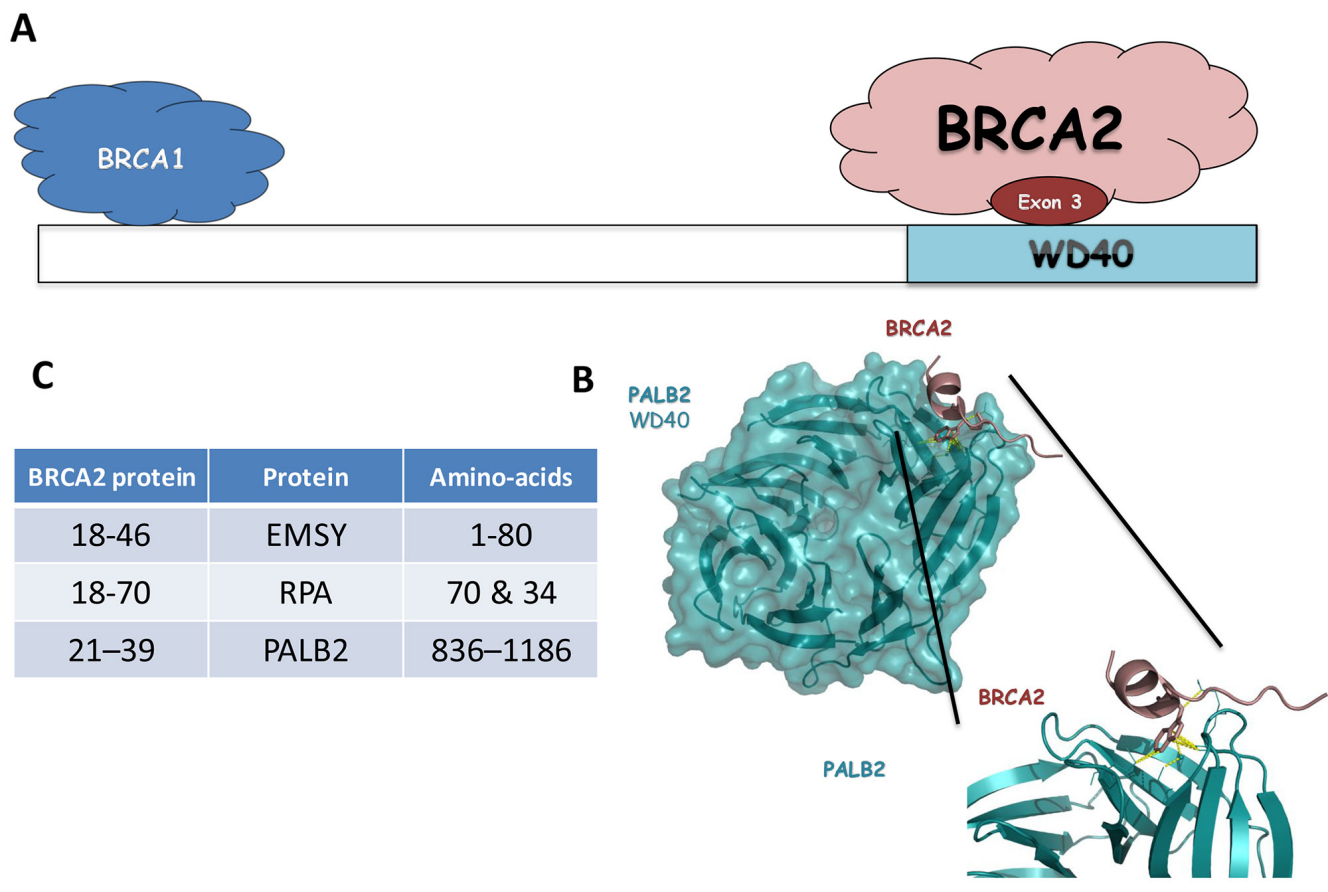
**(A)** Schematic representation of PALB2 domains and binding sites for its interacting partners (BRCA1 and BRCA2). **(B)** Interaction site with WD40 domain of the PALB2 protein and the exon 3 domain of the BRCA2 protein. Ribbon representation of the BRCA2-PALB2 complex (pdb ID: 3EU7). Domain WD40 of PALB2 is colored cyan and BRCA2 exon 3 is colored dark red. **(C)** Table of the interaction aminoacids for the EMSY and PALB2 proteins with the exon 3 domain of the BRCA2 protein.

Our results suggest that this part of the protein is important for the DNA damage repair function of BRCA2. Among all the BRCA2 partners described above, an obvious candidate for the DNA repair defective function of the protein lacking exon 3 observed in this study is loss of the PALB2 binding site [24, 25, 40–43] (Figure 4). The region of interaction has been defined by crystallography for BRCA2 (amino acids 21–39 part of exon 3) and PALB2 (WD40 motifs, amino acids 836–1186 from exon 6 to exon 13, Figure 4B and 4C). The level of cancer risk associated to *PALB2* pathogenic variants is known to be low in comparison to *BRCA2* pathogenic variants, but enough to propose a clinical management of the familly contrary to other low risk genes, notably *CHEK2* 1100delC and *ATM* [44]. The importance of this interaction for the DNA repair function of BRCA2 has been highlighted in several studies [25, 45, 46]. In particular, Siaud et al showed that a truncated BRCA2 protein lacking the entire C-terminal DNA binding domain could partially restore the DSB repair function of BRCA2 only when the PALB2 binding site was intact. These results strongly suggest that PALB2 interaction is important for the DSB repair function of BRCA2. BRCA2 and PALB2 cooperate in other related functions, such as the G2/M checkpoint control upon DNA damage [47]. In addition, *in vitro*, BRCA2 and PALB2 cooperate in stimulating RAD51-mediated D-loop formation, a critical step in HR [48]. Furthermore, PALB2 is an integral component of the BRCA complex [49] in which PALB2 mediates the physical interaction between BRCA2 and the C-terminal region of BRCA1 (Figure 4A) [25, 50]. These three proteins also act together to protect stalled replication forks from excessive degradation [5]. It has recently been shown that both the RAD51 recruitment to DSBs and the replication fork protection function are impaired due to disruption of BRCA2-PALB2 interaction [45]. Based on the work on the truncated protein [46], the PALB2 binding site precludes the DSB function of BRCA2. Whether the replication protective function is also altered in the delta-exon 3 protein warrants further investigation.

The importance of the exon 3 and the effect on the risk in the absence of this domain should help to reconsider some interaction already described. Hugues-Davies et al. found the *EMSY* gene to be amplified in 13% of all breast cancers and 17% of ovarian cancers (20% of cases of high-grade serous ovarian cancer [51]). EMSY was shown to be capable of silencing the transcription activation potential of a BRCA2 protein domain encoded by exon 3. Like BRCA2, EMSY also relocates to double-strand break repair sites after DNA damage. These results reinforce the functional link between BRCA2 and EMSY. The BRCA2 inactivation through EMSY amplification might be important in the tumorigenesis of a substantial proportion of non-inherited sporadic breast cancer. Recent studies have shown that amplification of EMSY was also associated with other cancers such as prostate ans pancreatic cancers [52]. Overexpression of EMSY interferes with the potential activation domain of BRCA2 encoded by exon 3 decreasing BRCA2 activity and resulting in a genomic instability phenotype as seen in BRCA2 deficient cells [22, 53]. The absence of the exon-3 domain then mimics this action of EMSY and increase the risks.

Other functions have been assigned to the exon-3 region., Smad3 have been described to be related to *BRCA2* both as modifier gene for the risk and with a direct functional interaction with exon 3 [54]. Another team was shown an interaction between BRCA2 (residues 18-70) and hRPA (polypeptides 70 and 34) [55]. This interaction BRCA2-hRPA is detected in the presence or absence of DNA binding by RPA. This is in contrast to a report that the p53-hRPA interaction is abrogated if RPA was prebound to DNA. For those proteins, their relevance for the functional effect observed in this study is less obvious and would deserve more investigations.

Furthermore, this approach, to combine information from several families with different genetic alterations resulting in the same final impact on the transcript provided the statistical power to calculate the odds of causality for seven variants. In principle, the same approach could be applied to other in-frame exons, in which deletion would be expected to lead to an internally deleted protein such as exons 10, 11, 12 [56], 17, 19, 26 and 27 of *BRCA2* or in other cancer susceptibility genes.

## CONCLUSIONS

This study demonstrates that full *BRCA2* exon 3 skipping is pathogenic, probably due to a defect in BRCA2 DNA damage repair function, and provides convincing evidence that variant alleles producing only transcripts lacking exon 3 should be considered to be pathogenic.

## MATERIALS AND METHODS

### Patient recruitment and consent

Variants under study were identified after genetic testing of patients reporting family history consistent with a high risk of carrying a *BRCA1* or *BRCA2* pathogenic variant. In France, eligibility for testing is defined by the French “UNICANCER Genetic Group” (UGG) and Inserm guidelines [57]. The families from ENIGMA were recruited from many different countries according to each country’s specific guidelines in place [31].

### Selected variants

Only variants suspected to result in complete deletion of *BRCA2* exon 3 at the transcriptional level were selected for this study. The transcriptional impact was based on both the literature and on the results of functional analysis performed in the various participating laboratories. Intronic variants were validated by a dedicated minigene assay.

We evaluated eight *BRCA2* variants resulting in a complete deletion of exon 3 (see Supplementary Table 1 and Table 1): c.68-?_316+?del, c.156_157insAlu, c.277_317-726delinsCCAT [36] c.316+1G>T, c.316+2T>C, c.316+4del, c.316+5G>A, c.316+5G>C. Data from 39 families were therefore collected from France, Portugal, Denmark and Sweden. Genotype and clinical data from variant carriers and relatives were obtained via diagnostic clinical testing (for laboratories in which a variant was considered to be pathogenic) or otherwise via ethically approved research testing following informed consent of the participants. In France, the latter included recruitment via the COVAR study (https://clinicaltrials.gov/ct2/show/results/NCT01689584). Co-segregation data were obtained for 293 patients from the 33 families in this study (194 confirmed as carriers of the variant under study).

### Cell-based minigene splicing assay

In order to evaluate the impact of the selected *BRCA2* exon 3 variants on RNA splicing, we performed a functional assay based on the comparative analysis of the splicing pattern of wild-type (WT) and mutant reporter minigenes, as follows. Minigenes were prepared by using the pCAS2 vector [58, 59]. First, WT and mutant *BRCA2* genomic fragments c.68-165_c.316+225 (*BRCA2* exon 3 and part of the flanking introns) were PCR-amplified from patient genomic DNA by using forward primer B2Ex3_Bam-F (5′- GACCGGATCCTTCGCAAGAGAATGGATTAATG-3′) and reverse primer B2Ex3_Mlu-R (5′- GACCACGCGTGGAGGGATGAAAGAGAACATTTAC-3′). Because patient’s DNA was not available for c.316+1G>T, we prepared the mutant DNA segment by site-directed mutagenesis using the two-stage overlap extension PCR method [Ho et al, 1989] and the pCAS2-BRCA2e3.WT construct as a template. After digestion with BamHI and MluI, the PCR products were inserted into the cloning sites of the reporter plasmid pCAS2, yielding the three-exon hybrid minigenes pCAS2-BRCA2e3. All constructs were sequenced to ensure that no unwanted mutations had been introduced into the inserts during the PCR or cloning process. Next, WT and mutant minigenes (400 ng/well) were transfected in parallel into HeLa cells grown at ∼70% confluence in 12-well plates using the FuGENE 6 transfection reagent (Roche Applied Science). HeLa cells obtained from ATCC were cultivated in Dulbecco’s modified Eagle medium (Life Technologies) supplemented with 10% fetal calf serum in a 5% CO2 atmosphere at 37°C. Twenty-four hours later, total RNA was extracted using the NucleoSpin RNA II kit (Macherey Nagel) according to the manufacturer’s instructions, and the minigenes’ transcripts were analysed by semi-quantitative RT-PCR (30 cycles of amplification) in a 25 μl reaction volume by using the OneStep RT-PCR kit (Qiagen), 200 ng total RNA and minigene specific primers (KOI-F 5′-TGACGTCGCCGCCCATCAC-3′ and pCAS2R 5′-ATTGGTTGTTGAGTTGGTTGTC-3′). RT-PCR products were separated by electrophoresis on 2.5% agarose gels containing ethidium bromide and visualized by exposure to ultraviolet light under saturating conditions using the Gel Doc XR image acquisition system (Bio-Rad), followed by gel-purification and sanger sequencing for proper identification of the minigene’s transcripts. Finally, splicing events were quantitated by performing equivalent fluorescent RT-PCR reactions followed by capillary electrophoresis on an automated sequencer (Applied Biosystems) using 500 ROX™ Size Standard (Applied Biosystems) and computational analysis by using the GeneMapper v5.0 software (Applied Biosystems).

### Bioinformatics predictions of splicing alterations

For each of the selected intronic variants, in silico predictions of their potential effects on splice sites were obtained by using the following in silico tools: SpliceSiteFinder-like (SSF, http://www.interactive-biosoftware.com), MaxEntScan (MES, http://genes.mit.edu/burgelab/maxent/Xmaxentscan_scoreseq.html) [60], NNSplice (NNS), GeneSplicer (GS) and Human Splicing Finder (HSF, http://www.umd.be/HSF/) [61]. These algorithms were simultaneously interrogated by using the integrated software Alamut (Interactive Biosoftware, http://www.interactive-biosoftware.com).

### Segregation analyses, multifactorial likelihood analysis and calculation of posterior probability of pathogenicity

Segregation analysis can be used to determine an odds or likelihood ratio quantifying the probability that a variant is linked to breast and/or ovarian cancer more than expected by chance. In a family in which most individuals carrying a particular variant develop breast/ovarian cancer, there is a strong likelihood that the variant causes the cancer phenotype. For a given variant, the product of the individual Bayes factors over the relevant families generates a likelihood ratio that quantifies the association with the disease [62]. In addition to segregation information as a direct measure of disease causality in families, the overall evaluation of causality can be calculated using multifactorial likelihood analysis [63] that includes additional information. For each variant, the likelihood ratio derived from co-segregation analysis was combined with other sources of data (family history, pathology data, etc.) to generate an overall likelihood score of causality. Information on segregation was available for all other families, and likelihood ratios (LRs) based on tumor pathology were available for a subset of variants. Multifactorial analysis was conducted using the methods described in Walker et al [64] which incorporates likelihoods for segregation and tumor pathology [65]. As previously described [63], probabilities were derived for each component under the assumption that each factor was statistically independent, the individual likelihood ratios were multiplied to calculate an overall multifactorial likelihood ratio, and Bayes factor analysis was then used to calculate a posterior probability that the variant was pathogenic from the multifactorial likelihood ratio and the prior probability. Given the knowledge that all variants resulted in the same aberration at the molecular level, and fact that the research question was to assess the clinical relevance of this molecular aberration (whether or not this aberration is pathogenic), all variants were assigned a prior probability of 0.5 [66, 67].

Variants were classified according to the criteria defined by Plon et al. [33], namely: Class 1: not pathogenic posterior probability (pp) 0.001; class 2: likely not pathogenic pp 0.001–0.049; Class 3: uncertain pp 0.05–0.949; Class 4: likely pathogenic pp 0.95–0.99; Class 5: pathogenic pp 0.99.

### Clonogenic survival assay

Cloning of the deletion of exon 3 from BRCA2 into GFP-MBP-BRCA2 was obtained by PCR from a fragment synthesized from a patient carrying the deletion using the following primers: 5’ TAACCGGTACCCAGCGGCCGCCCTATTGGATCCAAAGAG 3’ and 5’ CATATCAGGATCCACCTCAGCTCCTAGAC 3’. The insert was cloned into NotI and BbvCI sites of the GFP-MBP-BRCA2. All constructs were verified by DNA sequencing.

*Brca2*-deficient hamster cells (VC8) were transfected with human GFP-MBP-tagged full-length BRCA2, BRCA2 Δ exon 3 or known pathogenic missense BRCA2 variant D2723H (c.8167G>C, exon 18) cDNA expression constructs using Turbofect (Thermo Fisher Scientific) according to the manufacturer’s specifications. Cell populations were selected in HAM’s F10 media (10% FBS) containing 1 mg/ml G418 (Sigma-Aldrich). After one week in selection media, the cell population was seeded for clonogenic survival assay, incubated overnight and treated with Mitomycin C (Sigma-Aldrich) at concentrations of 0.25, 0.5 and 1.0 μM in serum-free HAM’s F10 media. Cells were treated for 1 h with the indicated concentrations of MMC, then washed, trypsinized, serially diluted and seeded in triplicates into 6-well plates. After 9 days of culture the cells were washed and stained with 1% crystal violet. Plates were dried overnight and colonies were counted to determine the survival fraction for each MMC concentration.

### Immunodetection of BRCA2

To verify protein expression, a fraction of the cells was harvested and lysed in lysis buffer containing 50 mM Hepes (pH 7.5), 250 mM NaCl, 5 mM EDTA, 1% NP-40, 1 mM dithiothreitol (DTT), 1 mM PMSF, and Protease Inhibitor Cocktail (Roche). GFPMBP-BRCA2 protein was detected from GFP-trap (ChromoTek) immunoprecipitates by immunoblotting with GFP antibody (Sigma G1544, 1:500).

## SUPPLEMENTARY MATERIALS TABLES


