# Supplementary material for: Full in-frame exon 3 skipping of BRCA2 confers high risk of breast and/or ovarian cancer
Source: Oncotarget. 2018 Apr 3;9(25):17334–48. doi: 10.18632/oncotarget.24671 (PMC5915120; doi:10.18632/oncotarget.24671)
Supplement: Supplementary file 1 [file oncotarget-09-17334-s001.pdf]

Full in-frame exon 3 skipping of *BRCA2* confers high risk of breast and/or ovarian cancer

SUPPLEMENTARY MATERIALS

APPENDIX

M. Mathieu-Dramard- CHU Amiens, Amiens; O. Ingster- Centre Paul Papin, Angers; P. Gesta- Centre Hospitalier d'Angoulême, Angoulème; H. Dreyfus- Institut Sainte-Catherine, Avignon; M.A. Collonge-Rame, J.L. Bresson, C. Populaire- CHU Besançon, Besançon; M. Longy; E. Barouk-Simonet, V. Bubien; N. Sévenet, F. Bonnet, N. Jones- Institut Bergonié, Bordeaux; I. Mortemousque- CH Jacques Cœur, Bourges; S. Audebert-Bellanger- CHU de Brest, Brest; Pascaline B.; A. Hardouin, D. Vaur, S. Krieger, L. Castéra- Centre François Baclesse, Caen; S. Ferrer- Centre Hospitalier Hôtel Dieu, Chambery; N. Uhrhammer, Y.J. Bignon- Centre Jean Perrin, Clermont-Ferrand; L. Faivre-Olivier, S. El Chehadeh; S. Lizard- CHU de Dijon, Dijon; O; Béra- CHU de Fort de France, Martinique; D. Leroux, H. Dreyfus, A. Béchet- CHU de Grenoble, Grenoble; H. Ranjatoelina- CHU Sud Réunion, La Réunion; V. Layet- Hôpital Flaubert, Le Havre; C. Adenis; J.-P. Peyrat, F. Revillion- Centre Oscar Lambret, Lille; S. Lejeune, S. Manouvrier-Hanu- CHRU Lille, Lille; L. Vénat-Bouvet- CHU Dupuytren, Limoges; C. Lasset, V. Bonadona; S. Mazoyer; O. Sininilkova, M. Léone, N. Boutry-Kryza- Centre Léon Bérard, Lyon; S. Giraud- Hospices civils de Lyon, Lyon; H. Sobol, T. Noguchi, V. Bourdon, A. Remenieras, F. Eisinger- Institut Paoli-Calmettes, Marseille;H. Zattara- CHU La Timone, Marseille; I. Coupier; J.-M. Rey, P.-O. Harmand- CHU Arnaud de Villeneuve, Montpelier; E. Luporsi- Centre Alexis Vautrin, Nancy; M. Bronner, J. Sokolowska-Gillois, P. Jonveaux, C. Philippe- CHU Nancy, Nancy; C. Delnatte, V. Guibert, S. Bézieau, E. Cauchin- Centre René Gauducheau, Nantes; A. Lortholary- Centre Catherine de Sienne, Nantes; V. Mari; M. Frenay- Centre Antoine Lacassagne, Nice; J. Chiesa- CHRU Caremeau, Nîmes; E. Rouleau, C. Lefol, R. Lidereau; V. Caux-Moncoutier, L. Golmard, C. Houdayer, B. Buecher, M. Gauthier-Villars, M. Belotti, A. Depauw, S. Demontety, D. Stoppa-Lyonnet-Institut Curie, Paris; F. Coulet, F. Soubrier, C. Colas, A. Fajac, M. Warcoin- Groupe Hospitalier Pitié-Salpêtrière, Paris; P.-L. Puig-HEGP, Paris; M. Lackmy-Port-Lis- CHU de Pointe à Pitre, Guadeloupe; A.-Marie Savoye; C. Delvincourt, O. Beaudoux- Institut Jean Godinot, Reims; Dominique Gaillard; C. Poirsier; M. Mozelle-Nivoix- CHU de Reims, Reims; C. Dugast; C. Abadie- Centre Eugène Marquis, Rennes; T. Frebourg, J. Tinat, A. Rossi, I. Thevenet- CHU de Rouen, Rouen; F. Prieur; M. Lebrun- CHU Saint-Etienne, Saint-Etienne; C. Noguès, E. Fourme, C. Sénéchal, A.-M. Birot, T. Kogut-Kubiak-Institut Curie, Saint-Cloud; J.-P. Fricker, H. Nehme-Schuster; D. Muller - Centre Paul Strauss, Strasbourg; C. Maugard- Hopital Universitaire de Strasbourg, Strasbourg; L. Gladieff; V. Feillel; C. Toulas- Institut Claudius Regaud, Toulouse; M. Mozelle- CHG Troyes, Troyes; O. Caron; M. Guillaud-Bataille, B. Bressac-De Paillerets- Institut Gustave Roussy, Villejuif

BRCA2 c.68-?_316+?del

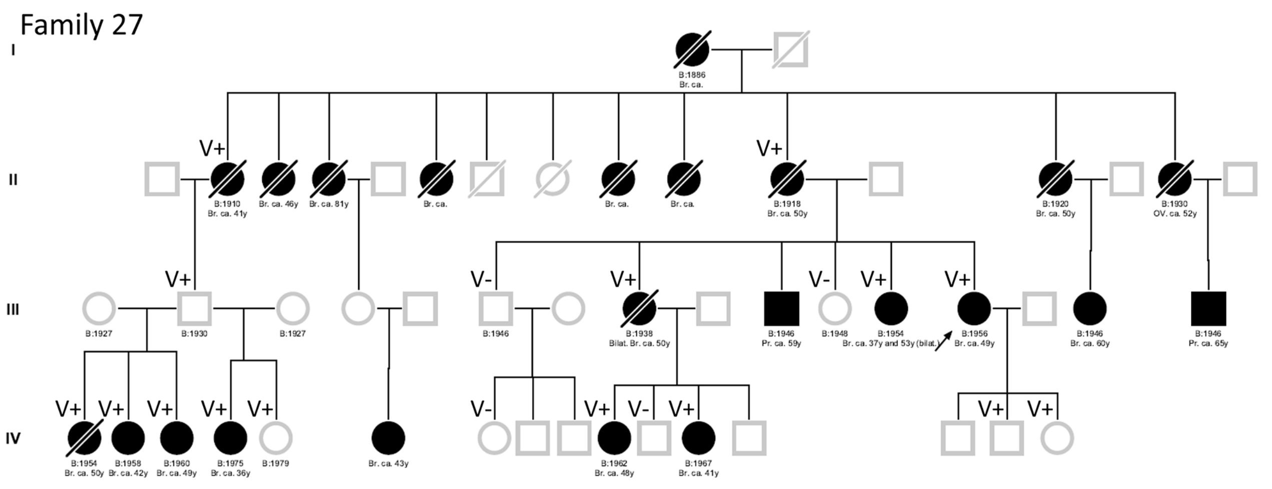

BRCA2 c.316+1G>T

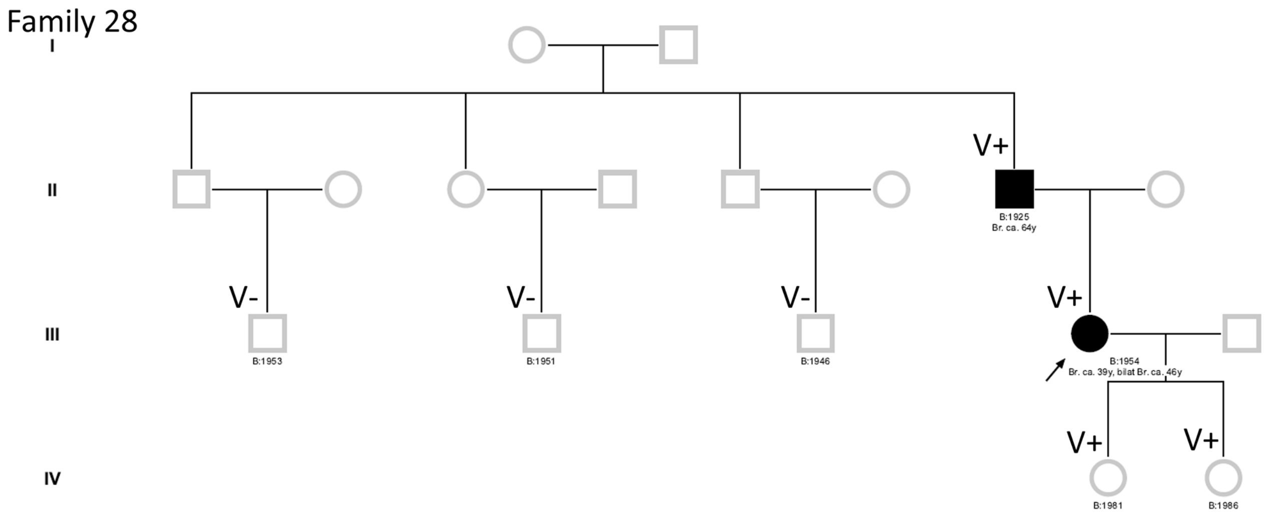

BRCA2 c.316+4del

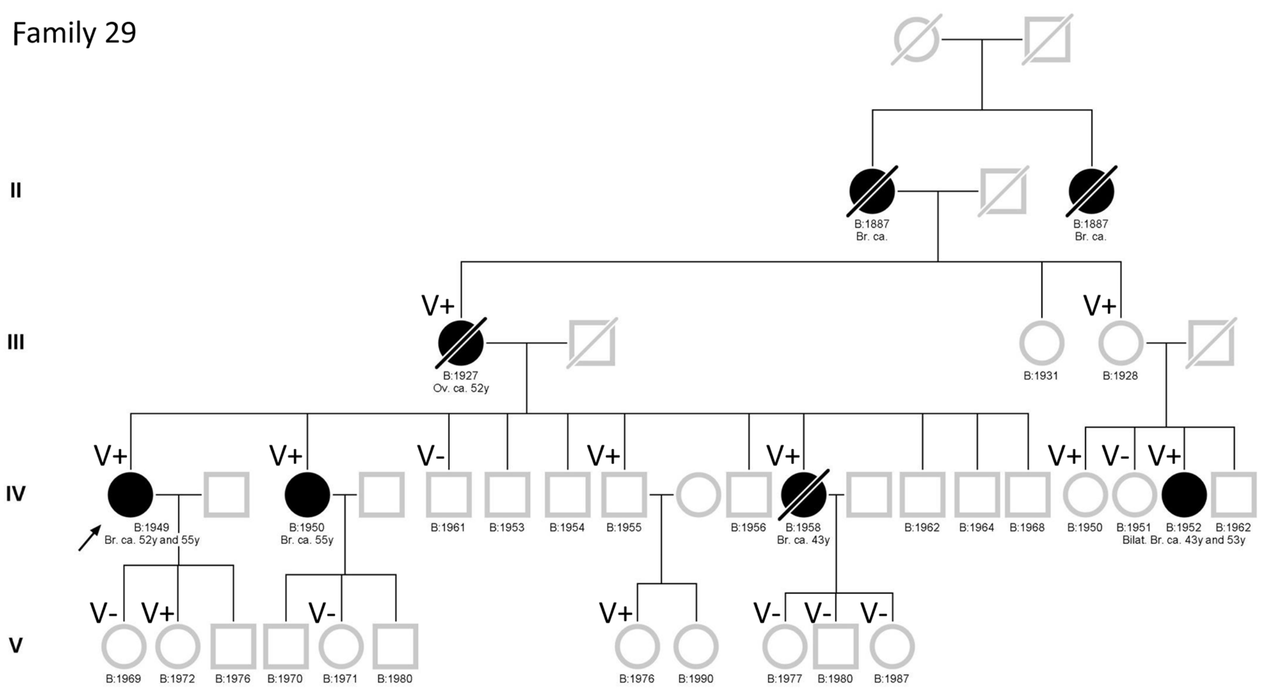

BRCA2 c.316+5G>A

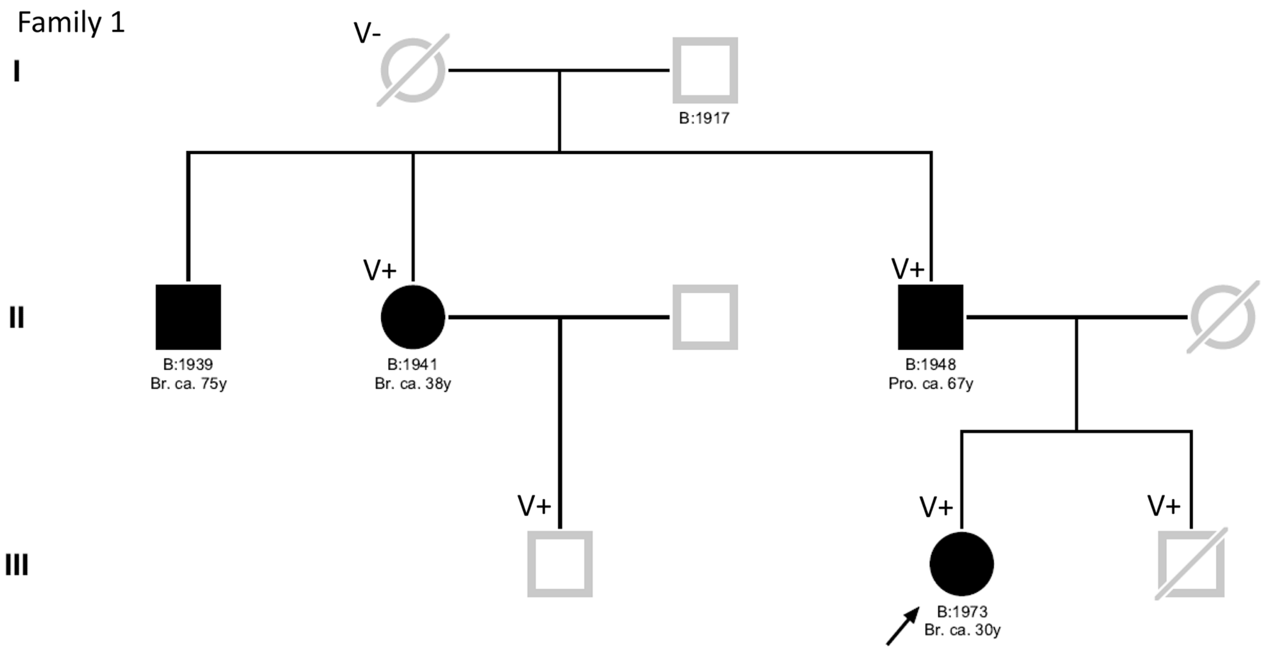

BRCA2 c.316+5G>C

Family 2

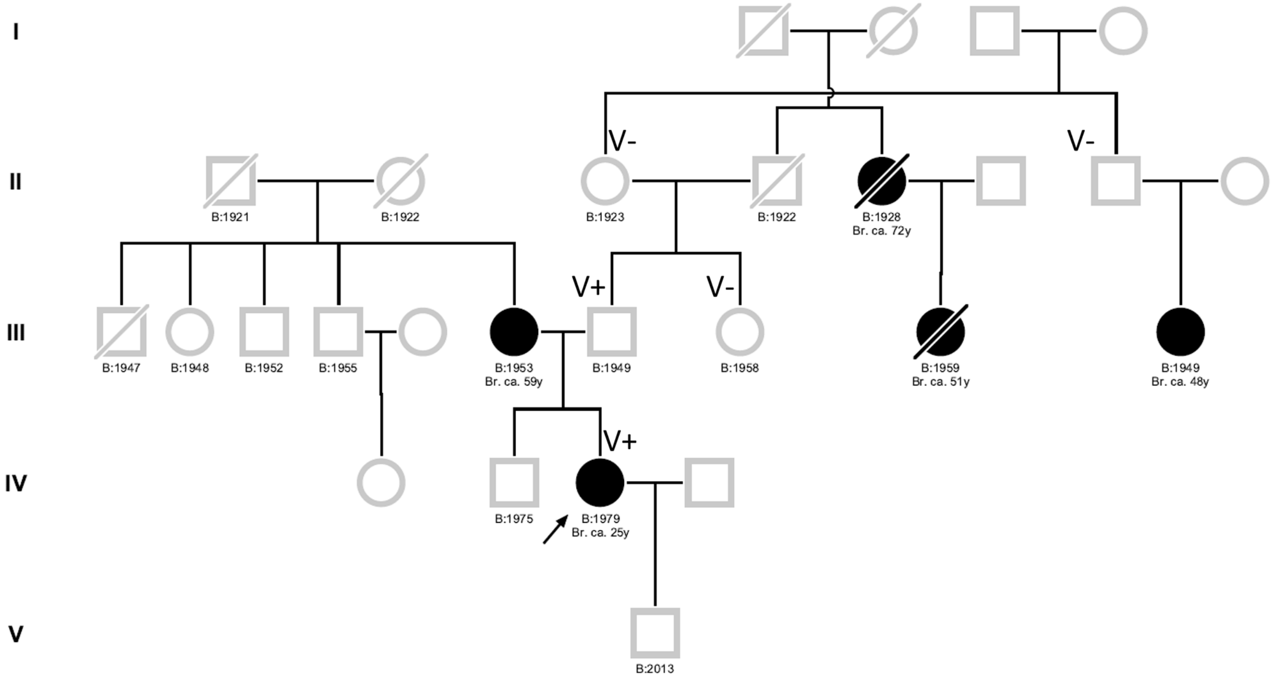

BRCA2 c.316+5G>C

Family 3

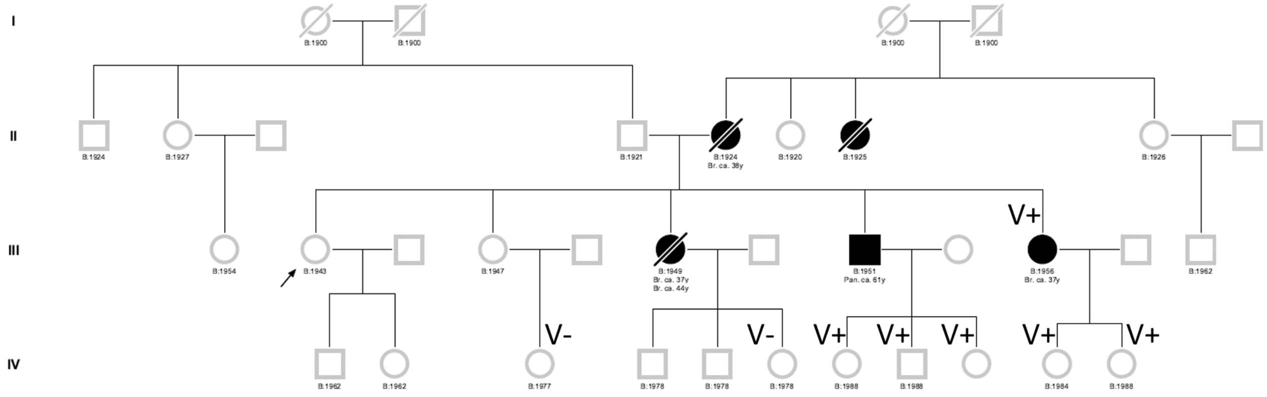

BRCA2 c.316+5G>C

Family 4

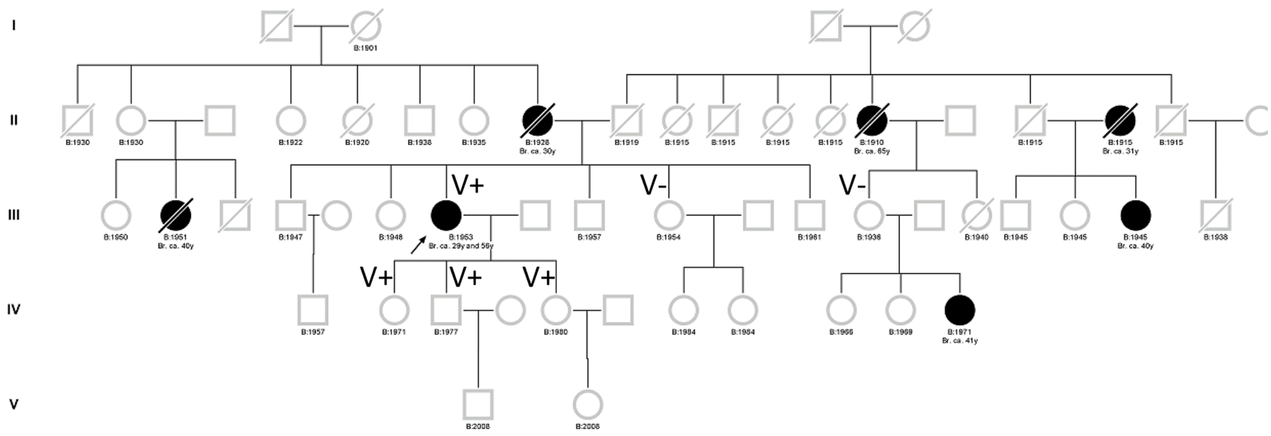

BRCA2 c.316+5G>C

Family 6

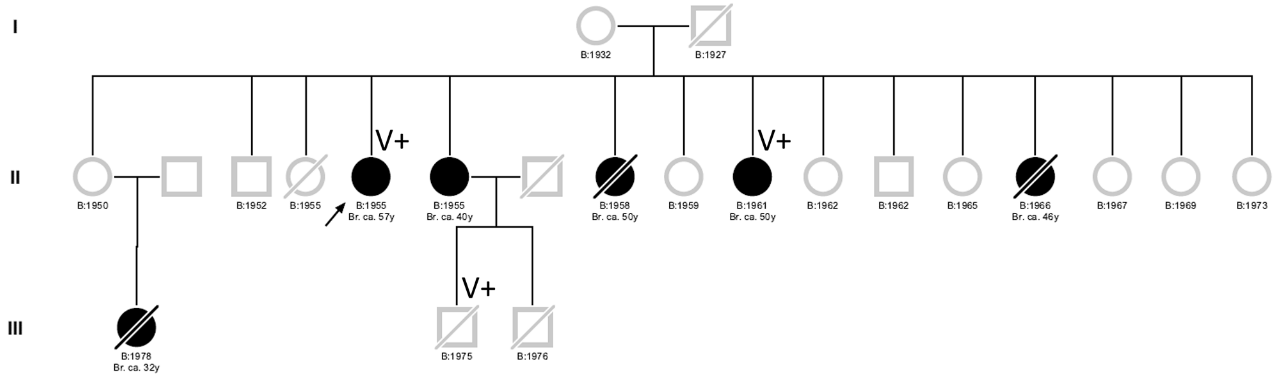

BRCA2 c.316+5G>C

Family 7

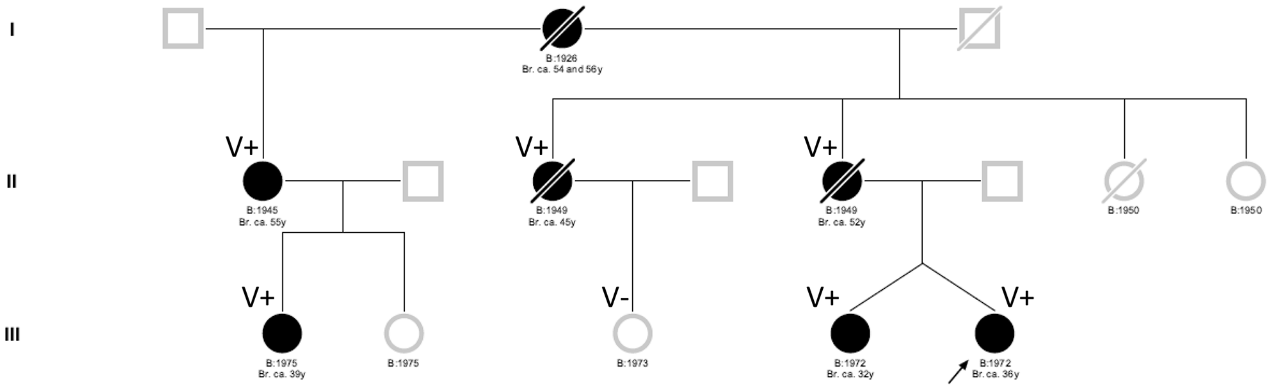

BRCA2 c.316+5G>C

Family 30

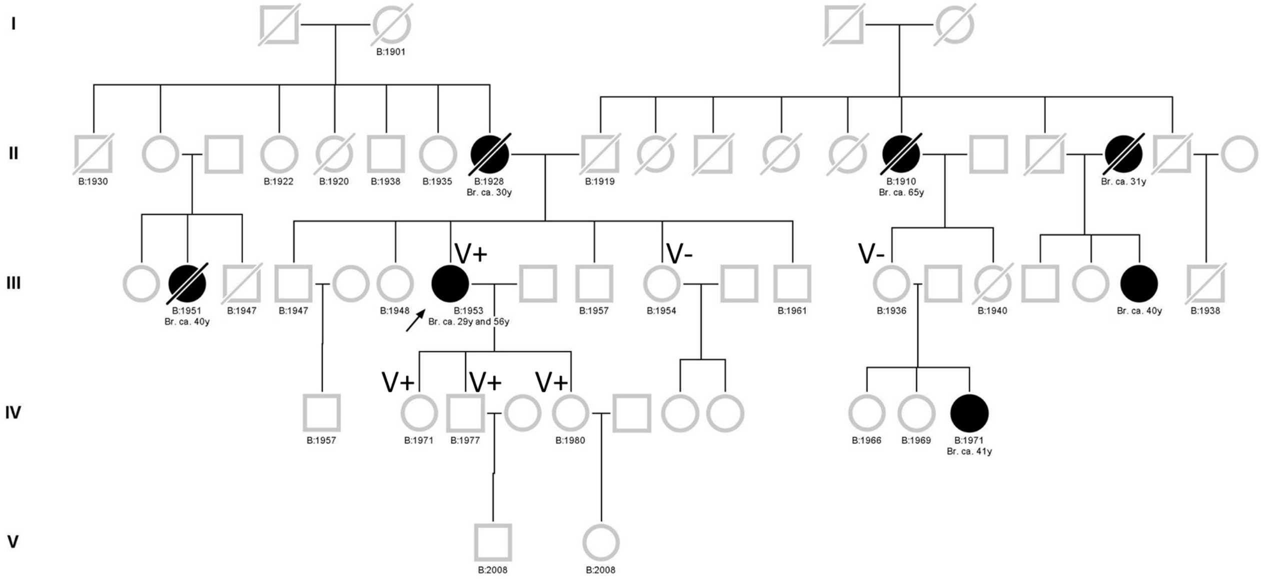

BRCA2 c.316+5G>C

Family 31

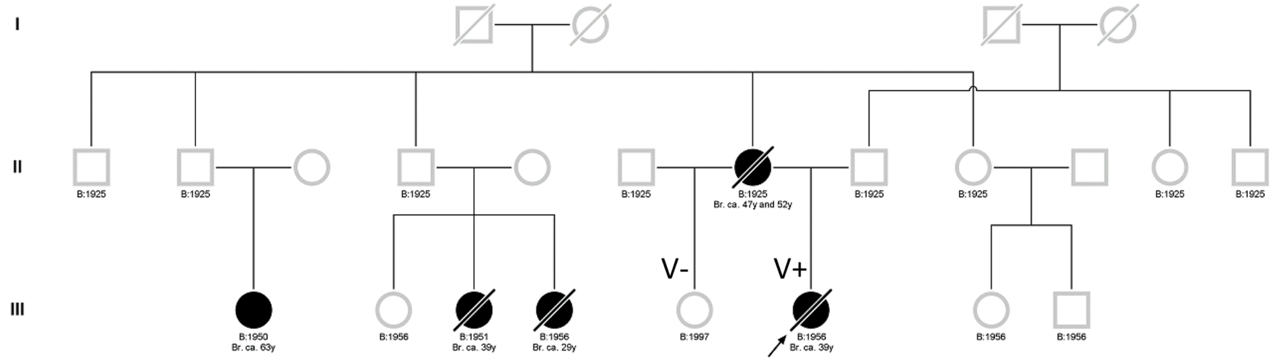

BRCA2 c.316+5G>C

Family 32

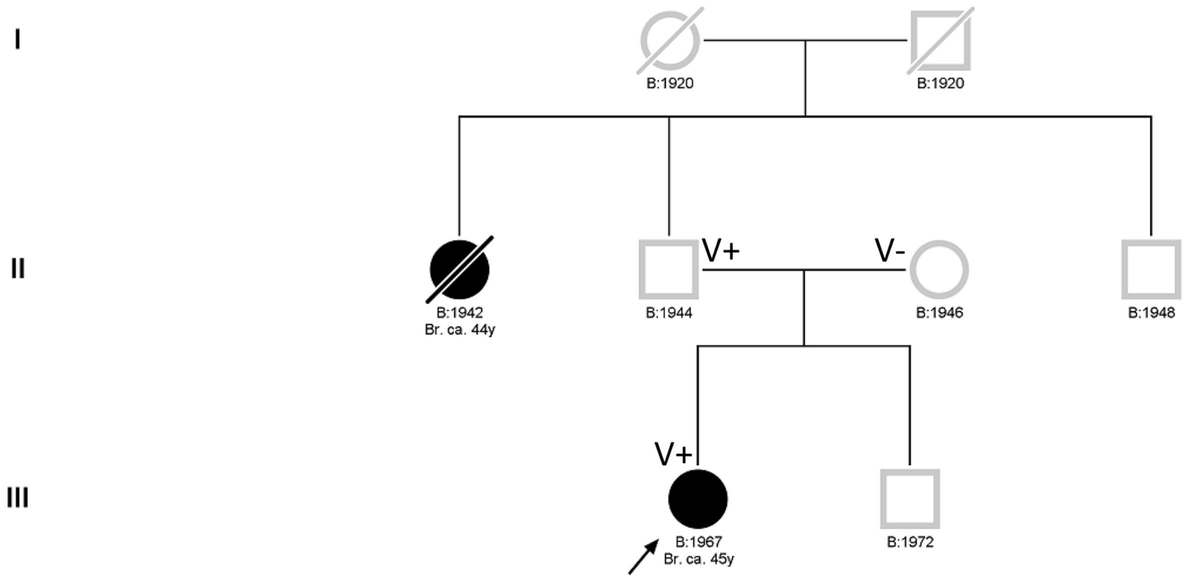

BRCA2 c.316+5G>C

Family 33

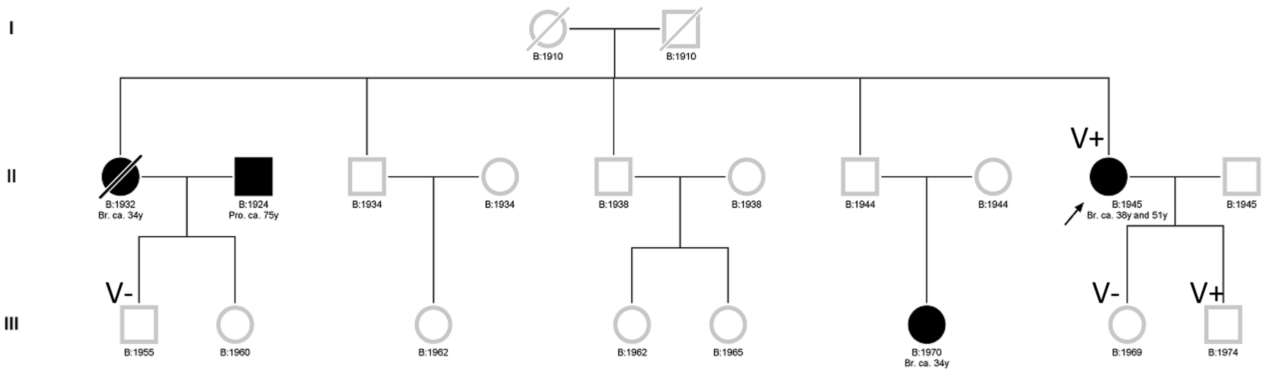

BRCA2 c.156_157insAlu

Family 9

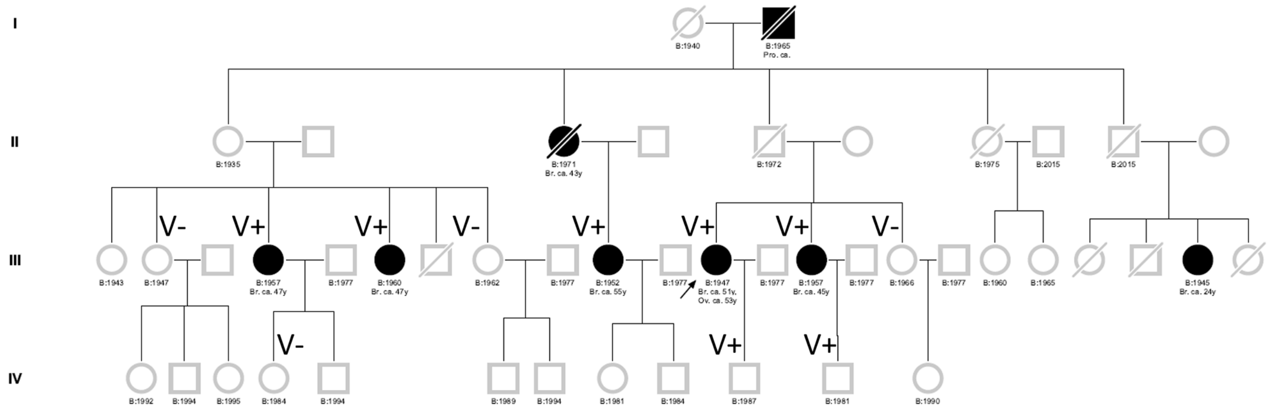

BRCA2 c.156_157insAlu

Family 10

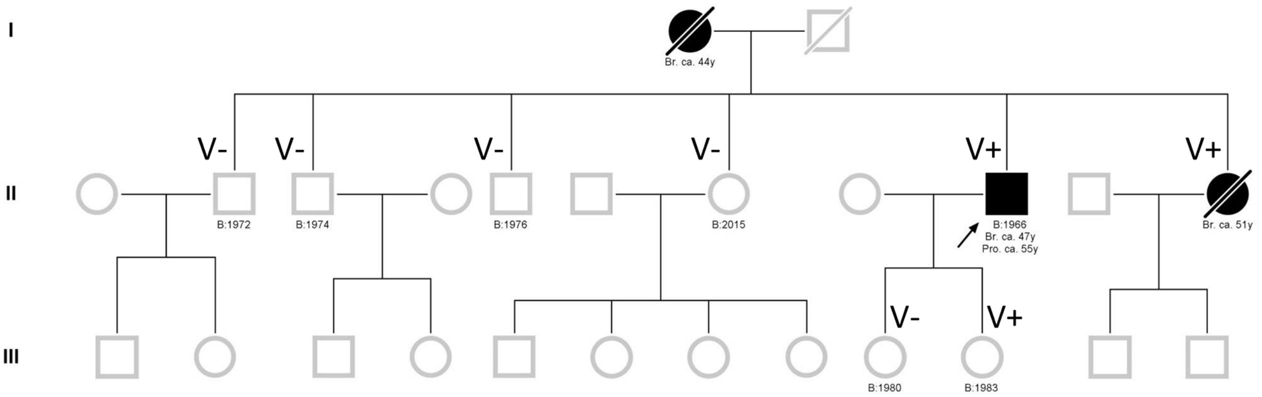

BRCA2 c.156_157insAlu

Family 11

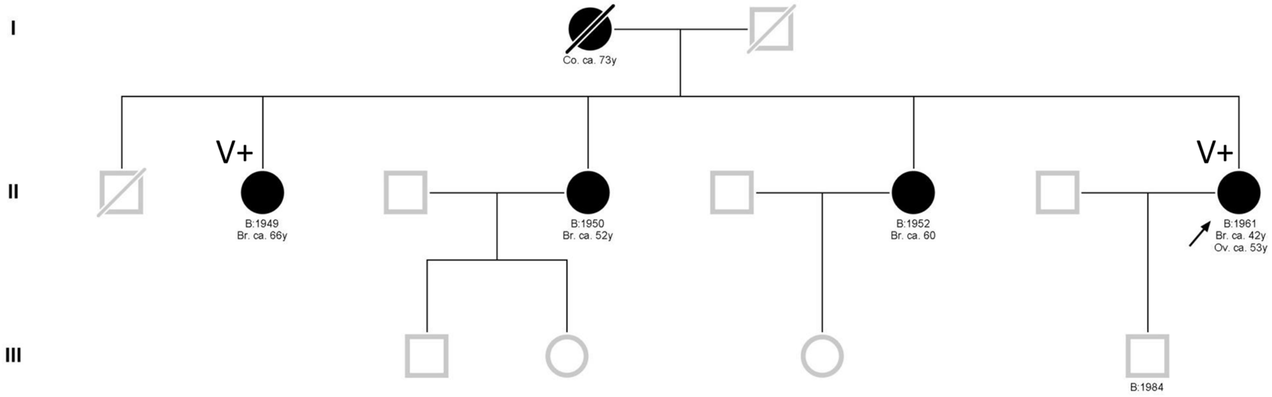

BRCA2 c.156_157insAlu

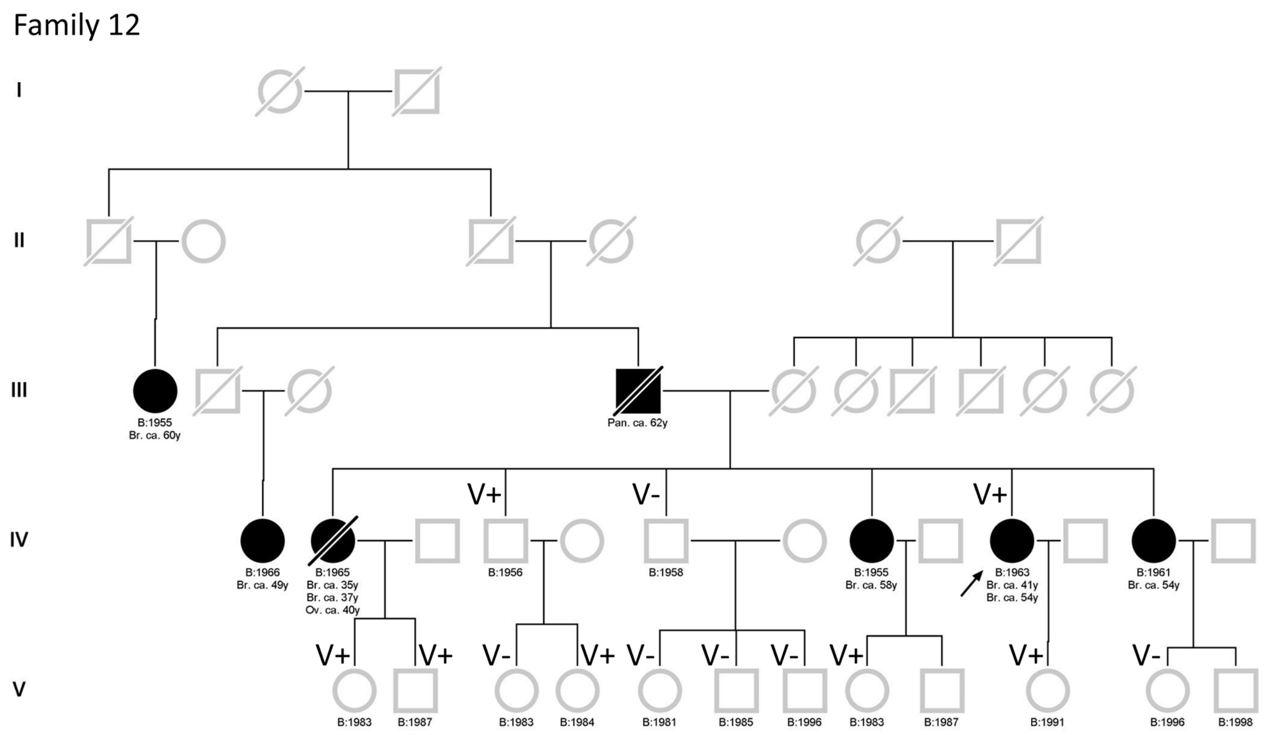

BRCA2 c.156_157insAlu

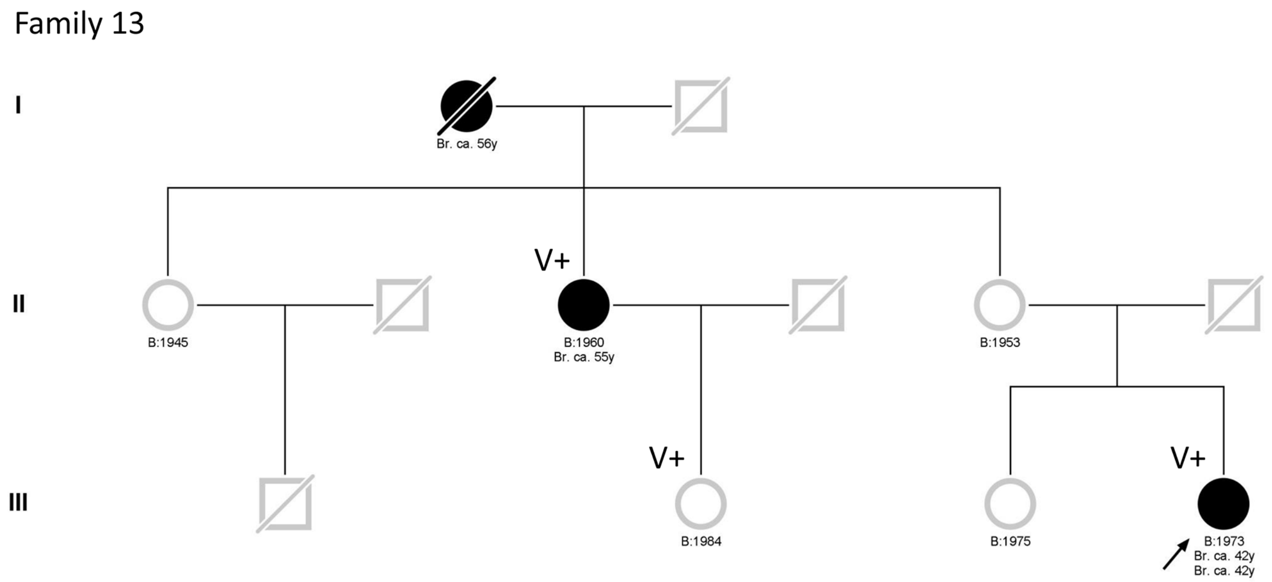

BRCA2 c.156_157insAlu

Family 14

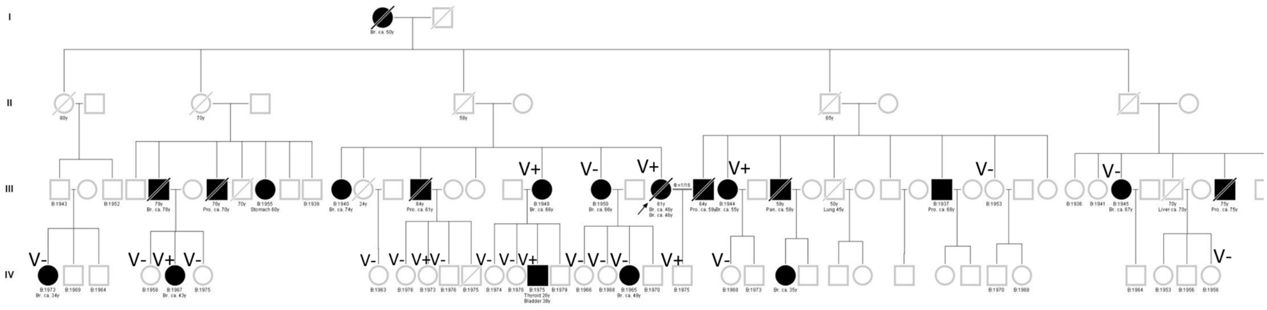

BRCA2 c.156_157insAlu

Family 15

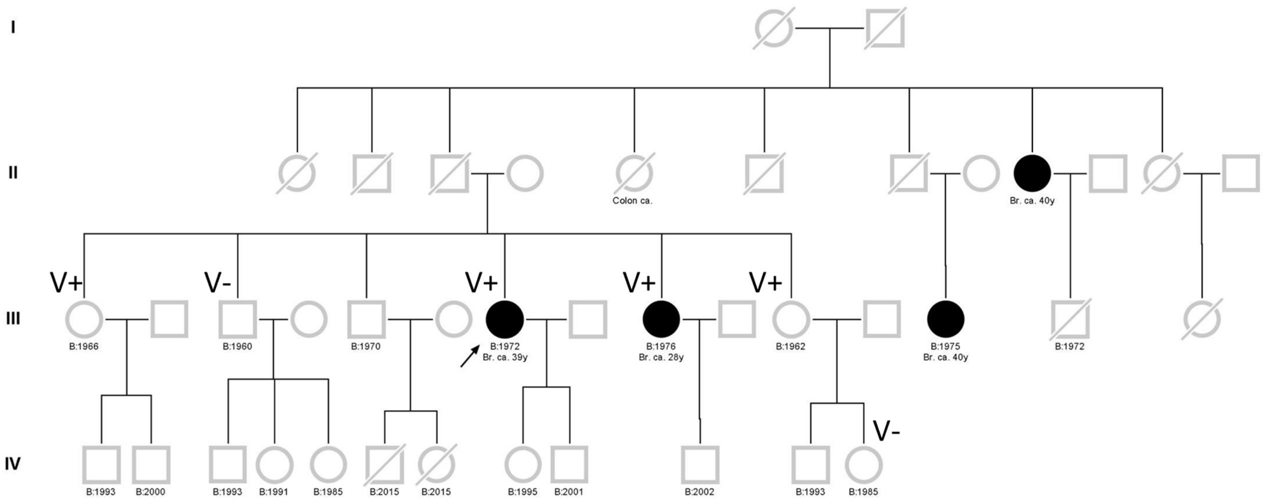

BRCA2 c.156_157insAlu

Family 16

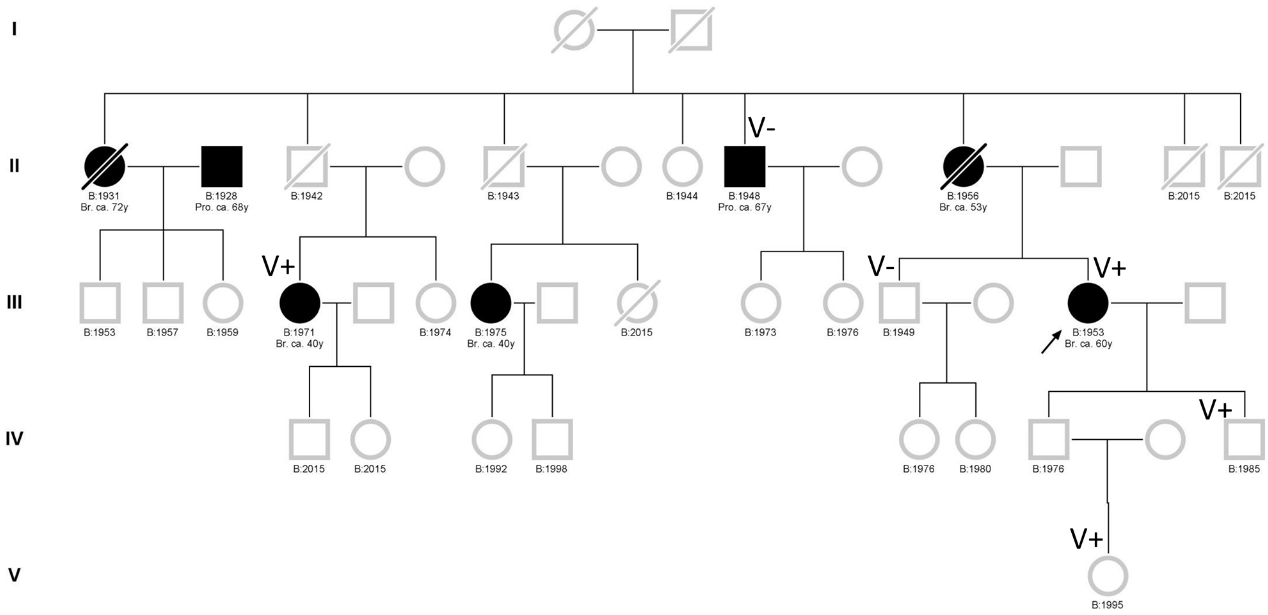

BRCA2 c.156_157insAlu

Family 17

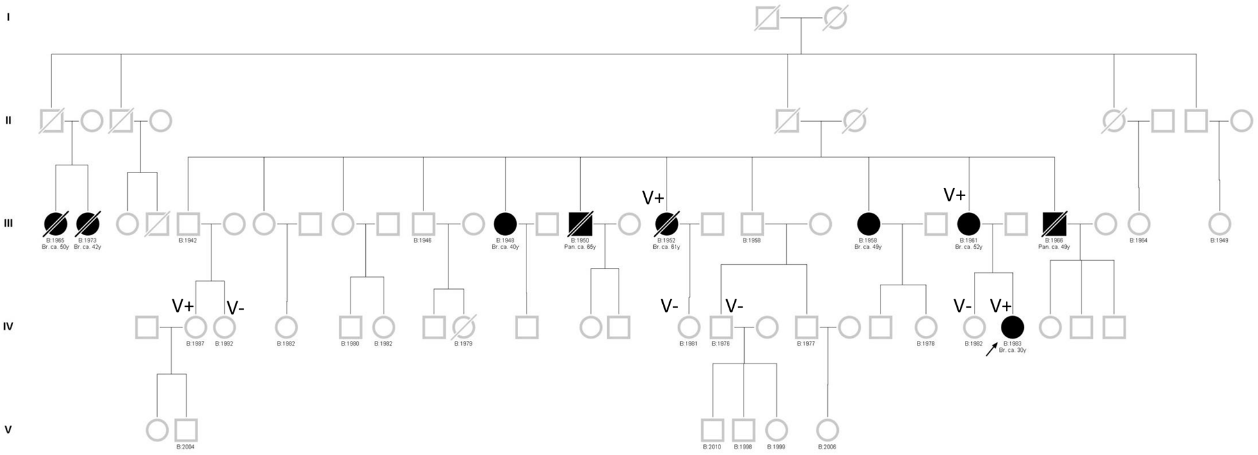

BRCA2 c.156_157insAlu

Family 18

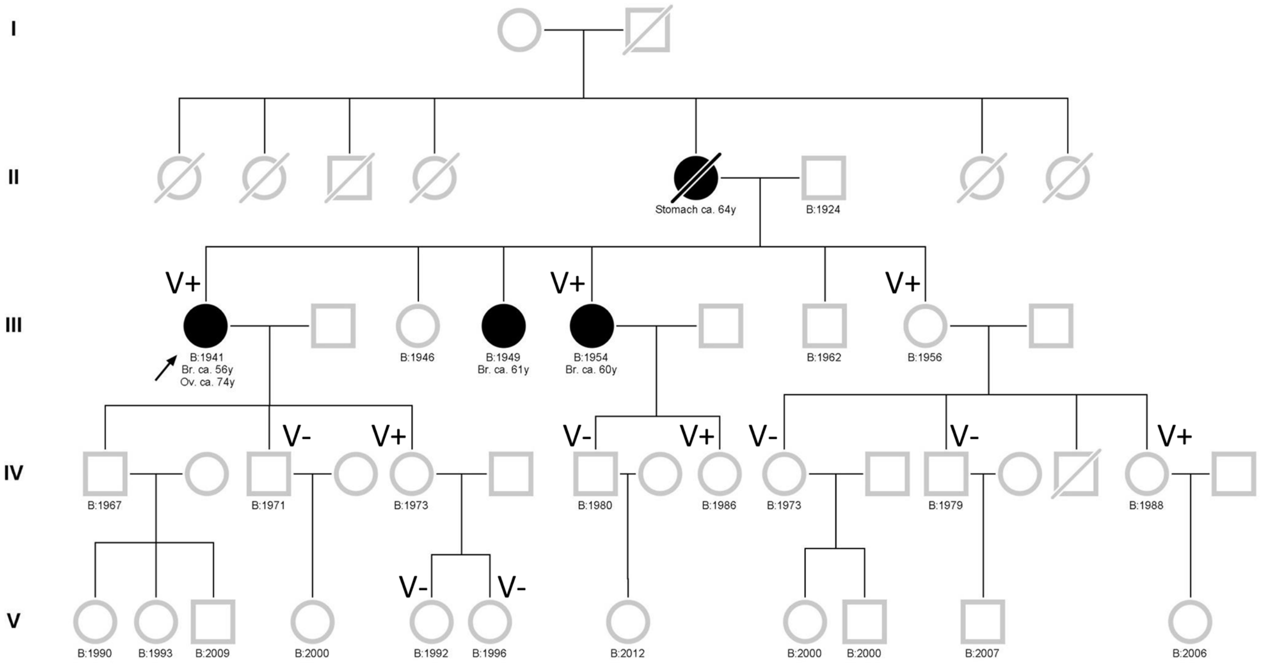

BRCA2 c.156_157insAlu

Family 19

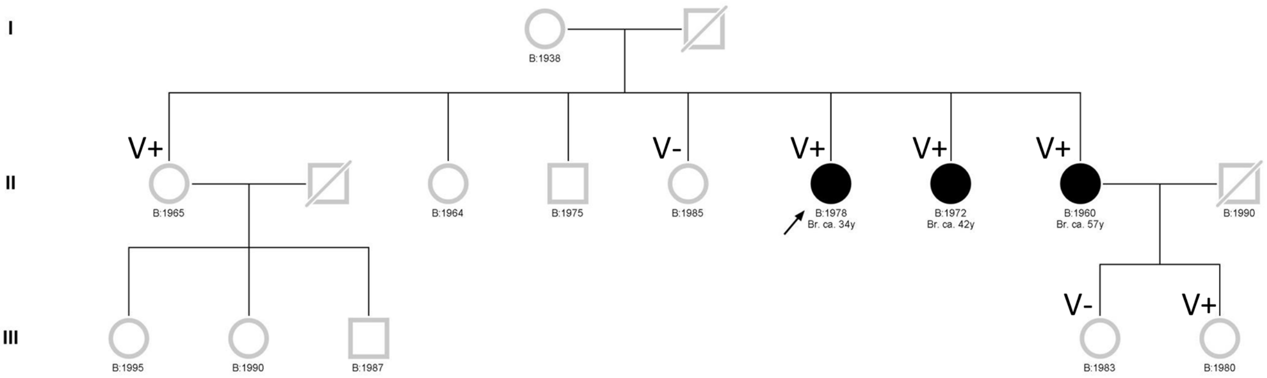

BRCA2 c.156_157insAlu

Family 20

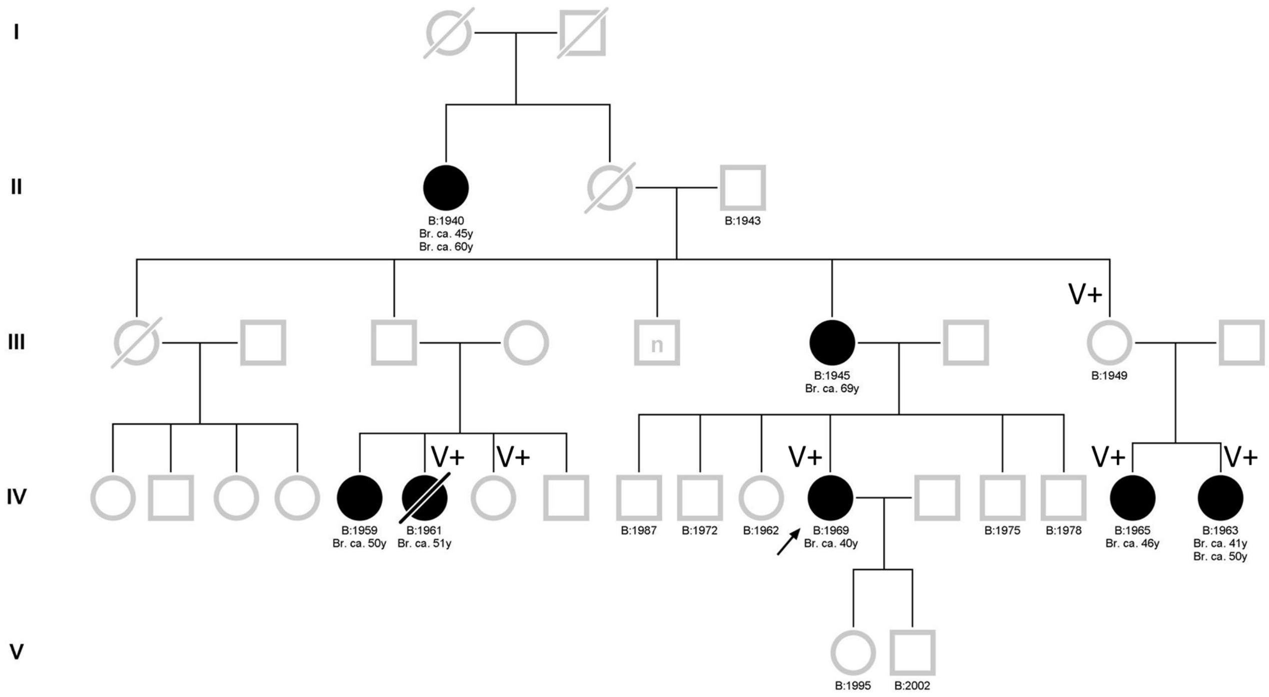

BRCA2 c.156_157insAlu

Family 21

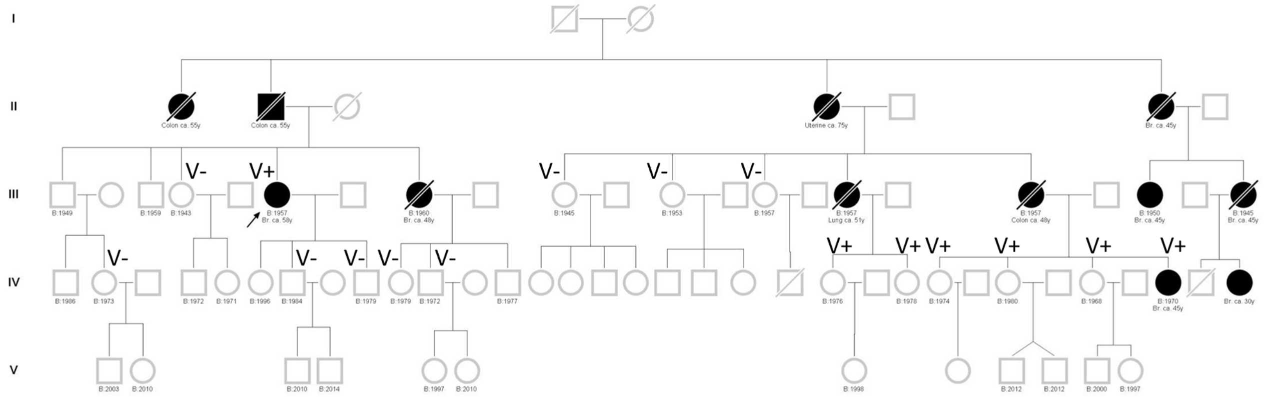

BRCA2 c.156_157insAlu

Family 22

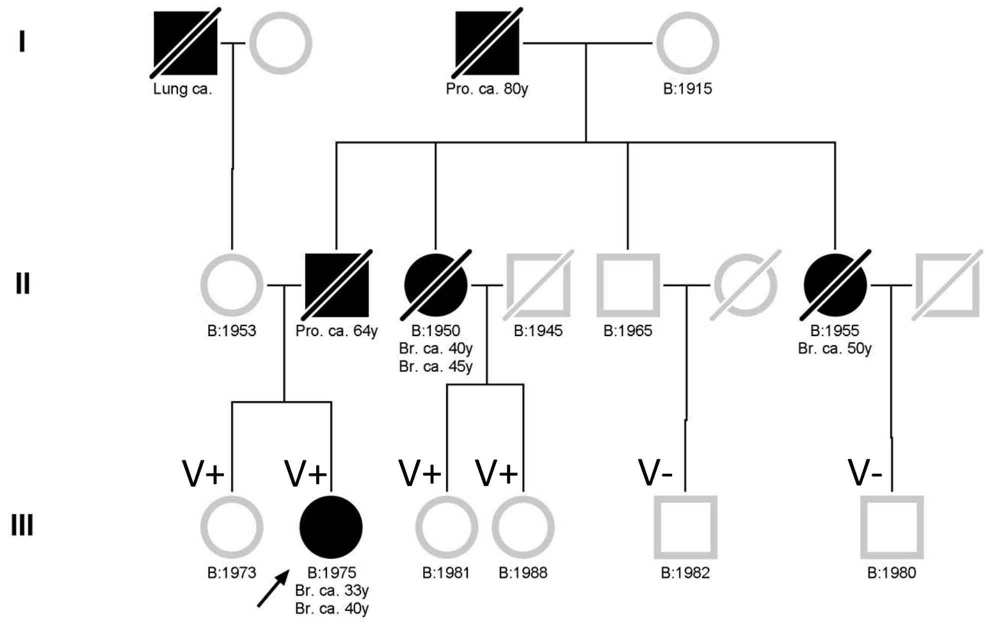

BRCA2 c.156_157insAlu

Family 23

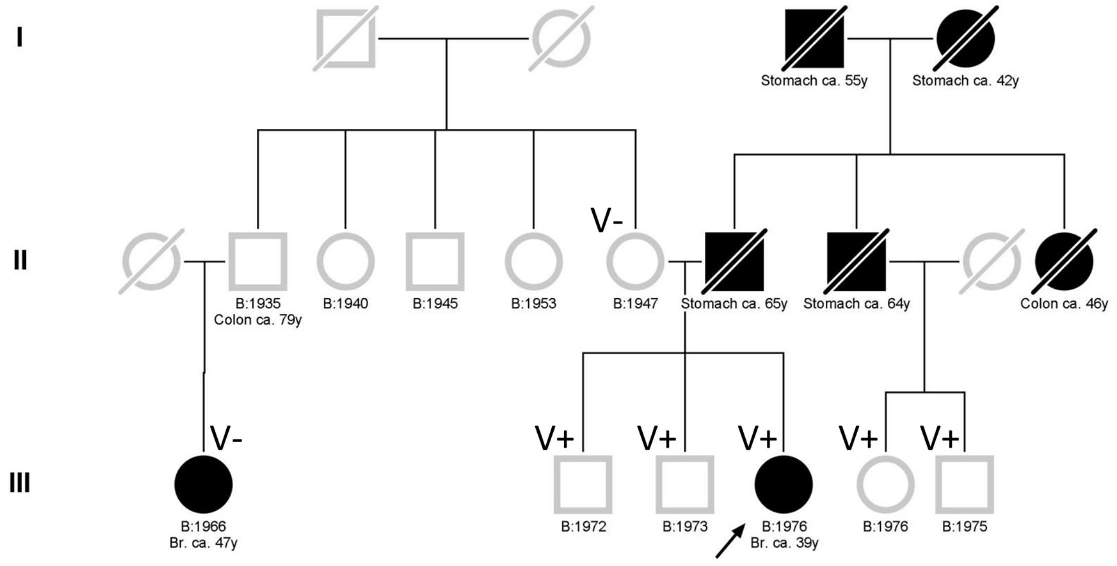

BRCA2 c.156_157insAlu

Family 25

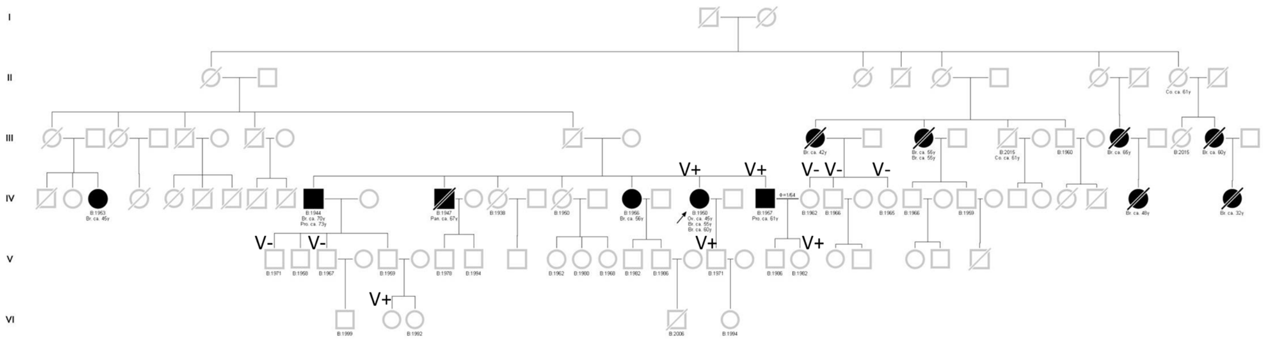

BRCA2 c.156_157insAlu

Family 26

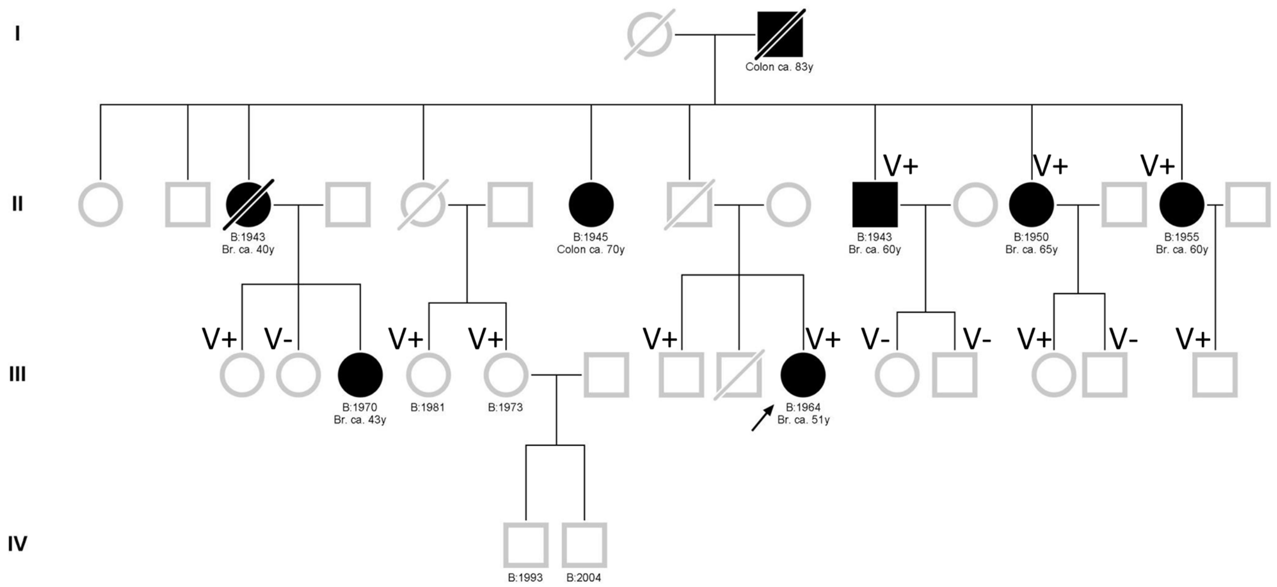

Supplementary Figure 1: Pedigrees of all families with co-segregation data of this study. Circles indicate females and squares indicate males, in bold relatives with mutated genetic status known but not affected. Slashes indicate death. The proband is indicated by an arrow. Current ages or age at death and age at cancer diagnosis are listed below each individual, together with genetic status when known.

Supplementary Table 1A: *BRCA2* variants analyzed in this study

BRCA2 nucleotide variant (HGVS DNA nomenclature c.)	RNA data	
	**Patients' RNA data (reference)**	**Minigene Splicing Assay data (reference)**
**c.68-?_316+?del**	Full exon 3 deletion as determined by RT-PCR electrophoresis and sequencing (Muller et al., 2011)	n.a.
**c.156_157insAlu**	Full exon 3 skipping as determined by allele-specific expression analysis (RT-PCR and sanger sequencing using SNP c.-26G>A) (Peixoto et al.,2009)<br>Exon 3 skipping as determined by RT-PCR electrophoresis and sequencing (Machado et al., 2007)	n.a.
**c.277_317-726delinsCCAT**	Full exon 3 skipping (Nordling et al., 1998)	n.a.
**c.316+1G>T**	n. d.	100% exon 3 skipping (this study)
**c.316+2T>C**	n. d.	97% exon 3 skipping (this study)
**c.316+4del (previously reported as c.316+3delA)**	Full exon 3 skipping as determined by allele-specific expression analysis (pyrosequecing of RT-PCR products using SNP c.-26G>A) (Muller et al., 2011)	100% exon 3 skipping (this study)
**c.316+5G>A**	Full exon 3 skipping as determined by allele-specific expression analysis (RT-PCR and sanger sequencing using SNP c.-26G>A) (Thomassen et al., 2012)	94% exon 3 skipping (this study)
**c.316+5G>C**	Full exon 3 skipping as determined by allele-specific expression analysis (mono-allelic RT-PCR using SNP c.-26G>A) (Bonnet et al., 2008)	Full exon 3 skipping (Bonnet et al., 2008)<br>95% exon 3 skipping (this study)

Supplementary Table 1B: Bioinformatics predictions of variant-induced splice site strength alterations

Splice site	Status	Splicing-dedicated *in silico* analysis											
		**SSF [0–100]**		**MES 3'ss [0–16] 5'ss [0–12]**		**NNS**		**GS**		**HSF-ss [0–100]**		**Frequence**	
		**Score**	**? (%)**	**Score**	**? (%)**	**Score**	**? (%)**	**Score**	**? (%)**	**Score**	**? (%)**	**Score**	**? (%)**
3' ss	WT	87.9	-	6.1	-	0.9	-	-	-	80.6	-	0.577	-
	c.68-7T>A	82.9	-6	4.6	-25	0.7	-22	-	-	78.5	-3	0.577	0
5' ss	WT	95.9	-	9.7	-	1.0	-	2.9	-	98.0	-	0.283	-
	c.316+1G>T	48.2	-50	0	-100	0	-100	0	-100	63.6	-35	0	-100
	c.316+2T>C	95.3	-1	0	-100	0	-100	0	-100	0	-100	0.019	-93
	c.316+4del	68.1	-29	3	-69	0.2	-20	0	-100	75.0	-24	0.001	-100
	c.316+5G>A	83.7	-13	6.6	-32	1.0	0	0	-100	85.9	-12	0.050	-82
	c.316+5G>C	83.2	-13	6.4	-34	1.0	0	0	-100	86.0	-12	0.019	-93

In silico analysis on splice sites for wild-type (WT) and BRCA2 intronic variants were performed by simultaneously interrogating 5 algorithms (SSF, MES, NNS, GS, and HSF) through the integrated software tool Alamut (Interactive Biosoftware). 3'ss and 5'ss refers to BRCA2 exon 3 reference 3' and 5' splice sites, respectively. SSF, SpliceSiteFinder-like; MES, MaxEntScan, NNS, NNSplice; GS, GeneSplicer; and HSF, Human Splicing Finder; The ranges of possible score values are indicated between brackets. Frequence of reference 3' and 5' splice sites refers to the frequence of the 6-mer or 9-mer found in constitutive human acceptor (3') or donor (5') sites, respectively. Δ, change relative to WT, expressed as a percentage.

Supplementary Table 2: Breast tumor pathology data from carriers of variants leading to full exon 3 skipping

Family	Cancer type	Age at diagnosis	Grade	Estrogen receptor status	Progesteron receptor status	HER2 status	LR pathology (Spurdle et al., 2014)*
**c.277_317-726delinsCCAT**							
Nordling et al., 1998 family	Breast	41	-	Positive	Negative	-	**1,15**
**c.316+5G>A**							
1	Breast	30	-	Positive	-	-	**1,15**
**c.316+5G>C**							
2	Breast	25	3	Negative	Negative	-	0,69
3	Breast	37	-	Positive	Positive	-	1,15
4	Breast	29	2	Positive	Negative	-	1,07
5	Breast	34	3	Positive	Positive	Negative	1,77
6	Breast	49	3	Positive	Positive	-	1,77
7	Breast	55	2	Positive	Positive	-	0,89
7	Breast	39	3	-	-	-	1,08
7	Breast	36	-	Positive	Positive	-	1,15
**Total**							**2,94**
**c.156_157insAlu**							
8	Breast	31	2	Positive	Positive	Positive	1,07
8	Breast	45	3	Negative	Negative	Positive	0,69
9	Breast	47	3	-	-	-	1,08
10	Breast	47	-	Positive	Positive	-	1,15
10	Breast	51	1	Positive	Positive	-	0,32
11	Breast	66	3	Positive	Positive	Negative	1,76
12	Breast	54	2	Positive	Negative	Negative	0,89
13	Breast	42	2	Positive	Positive	Negative	1,07
13	Breast	48	2	Positive	Positive	-	1,07
14	Breast	66	-	Positive	Positive	Positive	0,9
14	Breast	43	3	Positive	Positive	-	1,77
15	Breast	39	3	Positive	Positive	Negative	1,77
15	Breast	28	-	Positive	Positive	-	1,15
16	Breast	60	2	Positive	Positive	Negative	0,89
17	Breast	52	2	Positive	Negative	Negative	0,89
17	Breast	61	3	Negative	Negative	Negative	1,54
17	Breast	30	1	Positive	Positive	Negative	0,37
18	Breast	60	2	Positive	Negative	Negative	0,89
19	Breast	34	-	Positive	Positive	Negative	1,06
19	Breast	42	-	Negative	Positive	Negative	1,06
20	Breast	40	2	Positive	Positive	-	1,07
21	Breast	58	3	Positive	Negative	Negative	1,76
22	Breast	40	-	Positive	Positive	-	1,15
23	Breast	39	3	Positive	Positive	Negative	1,77
24	Breast	41	3	Positive	Positive	Positive	1,77
**Total**							**5,23**
